# Adaptaquin is selectively toxic to glioma stem cells through disruption of iron and cholesterol metabolism

**DOI:** 10.1002/1878-0261.70128

**Published:** 2025-09-21

**Authors:** Adrien M. Vaquié, Davod R. Shah, Eliane E. S. Brechbühl, Michael McNicholas, Zhaoyang Xu, John H. Stockley, Laura Morcom, Diana Gold Diaz, Gemma C. Girdler, Rachel V. Seear, Gabriel Balmus, Rajiv R. Ratan, Harry Bulstrode, James A. Nathan, Manav Pathania, Kevin M. Brindle, David H. Rowitch

**Affiliations:** 1 Wellcome-MRC Cambridge Stem Cell Institute, University of Cambridge, UK; 2 Department of Paediatrics, Biomedical Campus, University of Cambridge, UK; 3 Cancer Research-UK, Cambridge Research Institute, University of Cambridge, UK; 4 Department of Oncology, Early Cancer Institute, University of Cambridge, UK; 5 Cancer Research-UK, Children’s Brain Tumour Centre of Excellence, University of Cambridge, UK; 6 Department of Medicine, Cambridge Institute of Therapeutic Immunology and Infectious Disease (CITIID), University of Cambridge, UK; 7 Department of Clinical Neuroscience, UK Dementia Research Institute, University of Cambridge, UK; 8 Department of Molecular Neuroscience, Transylvanian Institute of Neuroscience, Cluj-Napoca, Romania; 9 Burke Neurological Institute and Brain and Mind Research Institute, Weill Cornell Medicine, White Plains, NY, USA; 10 Departments of Pediatrics and Neurosurgery, Cedars-Sinai Guerin Children’s, Los Angeles, CA, USA

**Keywords:** Adaptaquin, cholesterol, deferoxamine, glioma stem cells, iron, nononcogene addiction

## Abstract

Glioma stem cells (GSCs) from this aggressive brain cancer have been subject to nononcogene addiction therapeutic strategies, in particular targeting iron and cholesterol metabolic pathways. In this study, we show the small molecule Adaptaquin (AQ) has anti-GSC effects while sparing neurons, mature oligodendrocytes and astrocytes. Transcriptomic analysis of AQ-treated GSCs showed dramatic upregulation of iron transport genes and downregulation of genes involved in cholesterol biosynthesis. Indeed, we found cytotoxic effects of AQ on GSCs were potentiated when combined with the iron chelator deferoxamine (DFO). Notably, these effects were independent of PHD2 and HIF1α regulation, indicating a distinct pathway of action. Furthermore, we observed that the heme analogue, hemin, protects GSCs from AQ-mediated cell death, suggesting the presence of a functional heme transporter in GSCs, an observation confirmed by uptake of heme analogues. Importantly, we found that AQ treatment alone or in combination with iron chelators impaired cholesterol homeostasis in GSCs, leading to mitochondrial fragmentation and cell death. These findings suggest AQ in combination with iron chelators results in lethal disruption of cholesterol metabolism in glioma stem cells.

## Abbreviations

AQ: Adaptaquin
AQ/DFO: combination of 1 μM Adaptaquin and 10 μM deferoxamine
DFO: deferoxamine
DIPG: diffuse intrinsic pontine glioma
FTH: ferritin heavy chain
FTL: ferritin light chain
GBM: glioblastoma
GSC: glioma stem cell
HIF: hypoxia inducible factor
HMGCS1: 3-hydroxy-3-methylglutaryl-CoA synthase
INSIG1: insulin induced gene 1
MnPP: manganese (III) Protoporphyrin IX chloride
MRI: magnetic resonance Imaging
MSMO1: methyl monooxygenase 1
NSC: neural stem cell
OPC: oligodendrocyte precursor cell
PHDs: prolyl-hydroxylases
ROS: reactive oxygen species
SQLE: squalene epoxidase
TFRC: transferrin receptor
ZnMP: zinc (II) mesoporphyrin IX

## 1 Introduction

Glioblastoma (GBM) is a common and highly lethal brain tumour. It represents 50% of all primary brain and malignant central nervous system (CNS) tumours and has an age-adjusted incidence rate of 3.19 to 4.17 per 100 000 population per year [1]. GBM has a poor prognosis with an average survival between 10 and 20 months and only 5% of GBM patients reach a long-term survival of 5 years [2]. Standard therapy for GBM comprises surgical resection, radiotherapy and chemotherapy with Temozolomide (TMZ) [3]. While these therapies prolong the survival of patients, 85% of long-term glioblastoma survivors suffer neurological sequelae [4], because cranial radiation [5,6] or TMZ [7,8] also has neurodegenerative side effects [9]. Therefore, it would be useful to identify new classes of drugs that have antiglioma stem cell effects but also are neuron-sparing.

Glioblastomas have high levels of metabolic activity, proliferation and migration [10,11]. Patient tumours that exhibit elevated cholesterol uptake and iron metabolism are associated with unfavourable prognoses [12–14]. Indeed, this metabolic activity has been the subject of therapeutic strategies that targeted these pathways [14–16]. However, the precise role of iron trafficking in glioblastoma progression remains incompletely understood. Iron is essential for tumour cell proliferation, but its dysregulation can lead to toxic effects. For instance, iron contributes to oxidative stress via the Fenton reaction and promotes regulated cell death through ferroptosis [17].

While elevated HIF1α in GBM is associated with increased proliferation, survival, invasion and resistance to chemotherapy [18], inhibition of prolyl-hydroxylases (PHDs), preventing HIF1α degradation, can induce GBM cell death [19–21]. Adaptaquin (AQ), a hydroxy-quinoline inhibitor of PHDs, has been shown to have neuroprotective effects by inhibiting proferroptotic gene expression, protecting against glutamate-induce neuron death, and restoring mitochondrial function in neurons [22–24]. As AQ crosses the blood–brain barrier, it has been used in preclinical neuroprotective studies for neurodegenerative diseases. For instance, in a mouse brain haemorrhage model and Parkinson’s disease models, AQ prevents neuronal loss by suppressing proferroptotic genes [23–25].

We postulated that AQ could be representative of a potentially novel class of small molecules that could compromise GSC viability while sparing neurons. Here, we show that AQ selectively induces cell death in GSCs and proliferative cells while sparing mature neurons, oligodendrocytes and astrocytes. Transcriptomic analysis revealed that AQ dysregulates iron and cholesterol metabolism. Moreover, AQ toxicity is potentiated by iron chelation, leading to a significant reduction in cholesterol content. Finally, we show that AQ/DFO treatment compromises mitochondrial integrity and elevates reactive oxygen species (ROS) levels, further exacerbating GSC vulnerability. These results highlight the therapeutic potential of AQ in targeting GSC metabolism while sparing healthy neuronal populations.

## 2 Materials and methods

### 2.1 Human GSC and DIPG culture

Patient-derived GSCs and diffuse intrinsic pontine gliomas (DIPGs) were obtained from the Glioma Cellular Genetics Resource (gcgr.org.uk) and generated with ethics approval from the NHS Health Research Authority (East of Scotland Research Ethics Service, REC reference 15/ES/0094). All GSCs used in this study are *IDH* wild-type glioblastoma-derived cell lines. GSCs and DIPGs were plated as single cells in Laminin-coated plates (Bio-Techne Sales Corp, #344600501, Abingdon, UK) and maintained in GSC medium (DMEM/F12) (Sigma, #D8437, Gillingham, UK), 0.5 × B27 (Gibco, #17504-044, Waltham, MA, USA), 0.5 × N2 (Gibco, #17502-001), 1 × MEM NEAA (Gibco, #11140035), 0.012% BSA (Gibco, #15260-037), 0.1 mM 2-mercaptoethanol (Gibco, #31350-010), 1.45 g·L^−1^ D-glucose (Sigma, #G8644), penicillin–streptomycin supplemented with 10 ng·mL^−1^ EGF (Peprotech, #315-09, Waltham, MA, USA), 10 ng·mL^−1^ bFGF (Peprotech, #100-18b) and 1 μg·mL^−1^ Laminin. Cells were cultured in a humidified incubator at 37 °C, 5% CO_2_, 20% O_2_, and the medium was changed every 2–3 days. Cells were passaged after dissociation using Accutase (Life Technologies, #A1110501, Waltham, MA, USA). All experiments were performed with mycoplasma-free cells, and all cell lines have been authenticated within the past 3 years using single-cell 10× multiomics. Hypoxic conditions were achieved by reducing the oxygen concentration in the incubator (Binder, CB-260, Tuttlingen, Germany) to 1% or 5%. The incubator is designed with internal compartments or secondary doors to minimize oxygen influx during door openings, ensuring stable low-oxygen conditions.

### 2.2 Primary mouse neural stem cells (NSCs) isolation and culture

C57BL/6 mice (JAX, 000664, Bar Harbor, ME, USA) were housed, handled and euthanized in accordance with Cambridge University biomedical services guidelines and UK Home Office regulations, under the approved animal project license number PP4690795. Neonate C57BL/6 mice (unsexed P0–P3) were euthanized and decapitated in compliance with UK Home Office regulations. Brains were dissected and the sub-ventricular zone (SVZ) isolated in Hibernate-A (Thermo Scientific, #A1247501, Waltham, MA, USA) under a binocular microscope in a sterile hood. SVZ tissues were then incubated in Trypsin (Life Technologies, #15400054) for 5 min at 37 °C. Following incubation, the tissue lysate was triturated with a P1000 pipet for 1 to 2 min, centrifuged (300 ***g***, 5 min, RT) and resuspended in GSC media. Cell lysate was plated in Laminin-coated 24-well plate (Bio-Techne Sales Corp, #344600501) with a ratio of 3 wells per brain and incubated in a humidified incubator at 37 °C, 5% CO_2_, 20% O_2_. The next day, the medium from the 3 wells was triturated again with a P1000 pipet and plated into 3 new wells of a Laminin-coated 24-well plate, and new media was added to the first 3 wells. The media was replaced every other day with GSC media until good confluency is reached (50–70% confluency). Cells were then dissociated with Accutase and plated into a laminin-coated T25 flask for expansion. Purity of neural stem cells was measured by immunocytochemistry of Pax6, and cells were stored at −80 °C in 10% DMSO in GSC media. Neural stem cells (NSCs) were cultured in GSC media on a Laminin-coated plate with a media change every 2–3 days. Experiments were performed before passage 6.

### 2.3 Primary rat OPC, OL, astrocyte isolation and culture

Euthanized and decapitated Wistar rat (RGD_13508588) neonates (unsexed P4–P7) were obtained from Charles River Laboratories, UK (RRID:SCR_003792). Rat brains were dissected in Hibernate-A (Thermo Scientific, #A1247501) under a binocular microscope in a sterile hood. After removing olfactory bulbs and meninges, the brains were cut into small pieces using a sterile scalpel and incubated in 10 mL Hibernate-A containing 34 U·mL^−1^ papain solution (Lorne, #L5003126) and 20 μg·mL^−1^ DNAse1 (Sigma, #10104159001) for 40 min at 37 °C in an orbital shaker (0.1 g.). Brain lysates were then collected by centrifugation (5 min, 300 ***g***, RT) and resuspended in 8 mL neutralizing solution consisting of 2 mM sodium pyruvate (Sigma, #58636) and 1× B27 (Gibco, #17504044) in Hibernate-A. A single cell suspension was obtained by trituration with a fire-polished paster pipet. Cell lysates were passed through a 70-μm cell strainer (Scientific Laboratories Supplies, #352350, Nottingham, UK) into a filtered 22.5% Percoll solution (Sigma, #GE17544501) for a final volume of 50 mL. Centrifugation was carried out at 800 ***g*** for 20 min with no brakes to pellet cells while removing cellular and myelin debris. Supernatant was removed and cells were washed with ice-cold HBSS (Thermofisher, #14170112). Cells were then collected by centrifugation (300 ***g***, 5 min, RT) and resuspended in 1 mL of Red Blood Cell Lysing buffer (Sigma, #R7757) for 1 min. Cells were then washed with 12 mL HBSS and pelleted by centrifugation (300 ***g***, 5 min, RT). Astrocytes and oligodendrocyte precursor cells were isolated from this cell pellet as follows:

#### 2.3.1 Astrocytes isolation

Cells were resuspended in 80 μL ice-cold MWBi media (2 mM EDTA (Thermo Scientific, #15575020), 2 mM sodium pyruvate, 0.5% BSA (Thermofisher, #A23015), 25 μg·mL^−1^ insulin (Sigma, #I9278)) supplemented with 20 μL of anti-GLAST (ASCA-1) Microbeads (Miltenyi Biotec, #130095825, Bisley, UK). The mix was incubated for 10 min at 4 °C with gentle agitation. After incubation, 8 mL of MWBi media was added to the mix and cells were collected by centrifugation (300 ***g***, 5 min, RT). The cell pellet was then resuspended into 80 μL MBWi supplemented with 20 μL of Anti-Biotin Microbeads (Miltenyi Biotec, #130095825) and incubated for 15 min at 4 °C with gentle agitation. After incubation, 8 mL of MWBi media was added to the mix and cells were collected by centrifugation (300 ***g***, 5 min, RT) and resuspended in 2 mL of MWBi media. The cell suspension was loaded into a MACS LS column (Miltenyi Biotec, #130042401). GLAST positive cells were isolated in a MACS Multistrand (Miltenyi Biotec, #130042303) according to the manufacturer’s instructions and resuspended in Astrocyte media (1× B27, 25 μg·mL^−1^ insulin, 1.5 mM sodium pyruvate, 60 μg·mL^−1^ *N*/acetyl cysteine (Sigma, #A8199)), 1× SATO (100× SATO: 1.61 mg-mL^−1^ putrescin dihydrochloride (Sigma, #P5780), 4 μg·mL^−1^ sodium selenite (Sigma, #S526110), 60 μg·mL^−1^ progesterone (Sigma, #P8783), 41.25 mg·mL^−1^ bovine serum albumin (Sigma, #A4919), 5 mg·mL^−1^ apo-transferrin (Sigma, #T1147) in DMEM/F12 (Gibco, #11039021)), 5 ng·mL^−1^ HB-EGF (Peprotech, #10047) in DMEM/F12. Astrocytes were plated into poly-L-lysine coated plates and incubated in a humidified incubator at 37 °C, 5% CO_2_, 5% O_2_ for 4–-7 days. Cell purity was assessed for each isolation by immuno-cytochemistry of DAPI and GFAP.

#### 2.3.2 Oligodendrocyte precursor cells (OPC) isolation and oligodendrocyte maturation

Cells were resuspended in 500 μL ice-cold MWBi media supplemented with 1 μL anti-A2B5 (Sigma, #MAB312) and incubated for 30 min at 4 °C with gentle agitation. After incubation, 8 mL of MWBi was added and cells were collected by centrifugation (300 ***g***, 5 min, RT). Cell pellet was then resuspended into 80 μL MBWi supplemented with 20 μL of Anti-Mouse IgM Microbeads (Miltenyi Biotec, #130047301) and incubated 15 min at 4 °C with gentle agitation. After incubation, 8 mL of MWBi media was added to the mix and cells were collected by centrifugation (300 ***g***, 5 min, RT) and resuspended in 2 mL of MWBi media. The cell suspension was loaded into a MACS LS column. A2B5-positive cells were isolation in a MACS Multistrand according to the manufacturer’s instruction and resuspended in OPC media consisting in Base media (1 mM sodium pyruvate, 25 μg·mL^−1^ insulin, 60 μg·mL^−1^ *N*-acetyl cysteine, penicillin–streptomycin, 1× SATO in DMEM/F12) supplemented with 10 ng·mL^−1^ bFGF (PeproTech, #10018B500) and 10 ng·mL^−1^ PDGF-AA (PreproTech, #10013A100). OPC were plated into poly-D-lysin coated plates and incubated in humidified incubator at 37 °C, 5% CO_2_, 5% O_2_ in OPC media. Cell purity was assessed for each isolation by immuno-cytochemistry of DAPI and Olig2. For oligodendrocyte differentiation, OPC media was replaced with OL media (Base media supplemented with 40 ng·mL^−1^ T3 (Sigma, #T5516)) and cells were maintained for 7 days in OL media before drug treatments and immuno-cytochemistry.

### 2.4 Primary rat hippocampal neurons isolation and culture

Euthanized and decapitated Wistar rat (RGD_13508588) neonates (unsexed P0–P2) were obtained from Charles River Laboratories, UK (RRID:SCR_003792). Hippocamps were dissected in Hibernate-A (Thermo Scientific, #A1247501) under a binocular microscope in a sterile hood. Hippocampal tissue was then cut into small pieces using a sterile scalpel and incubated in Hibernate-A containing 34 U·mL^−1^ papain solution (Lorne, #L5003126) and 20 μg·mL^−1^ DNAse1 (Sigma, #10104159001) for 20 min at 37 °C with gentle agitation. Tissue lysates were then collected by centrifugation (5 min, 300 ***g***, RT) and resuspended in 3 mL neutralizing solution consisting of 2 mM sodium pyruvate (Sigma, #58636) and 1× B27 (Gibco, #17504044) in Hibernate-A. A single cell suspension was obtained by trituration with decreased size of fire-polished pasteur pipet. Then, the cell lysate was passed through a 40-μm cell strainer (Corning, #431750, Glendale, CA, USA) and plated in a 24-well plate for 3 min. Immediately after, the cell suspension was removed from the plate and a small aliquot was used to count cells on a hemocytometer. Cells were plated at a density of 100 000 cells per cm^2^ on a poly-D-lysine coated plate in Plating Media (10% Horse serum (Thermofisher, #26050088), 1× GlutaMAX, 2% B27, 20 mM HEPES pH 7.5 (Sigma, #H3375) in Neurobasal (Gibco, #21103-049)) and incubated in a humidified incubator at 37 °C, 5% CO_2_, 20% O_2_. The next day, media was replaced with Neuron media (2% B27, 1× GlutaMAX, 1× penicillin/streptomycin in Neurobasal) supplemented with 10 μM 5-fluoro-2^′^-deoxyuridine (FDU) (Sigma, #F0503) for 2 days. After 2 days, media was replaced with Neuron media without FDU for the following 2 days. Then, the media was replaced with Neuron media with FDU for 2 days. Finally, the cells were maintained in Neuron media without FDU for at least 10 days before drug treatment. Cell purity was assessed by morphology under a binocular microscope.

### 2.5 Human induced pluripotent stem cells (iPSC)-derived neurons

iPSC-NG2 cells [26,27] were dissociated into single cells with Accutase (Life Technologies, #A1110501) and plated at 25 000 cells per cm^2^ in a six-well plate precoated with Vitronectin (Stem Cell Technologies, #07180, Cambridge, UK) for 1 h and cultured at 37 °C in STEMFLEX media (Thermo Scientific, #A3349401) supplemented with 10 μM ROCK inhibitor Y-27632 (Tocris, #1254, Abingdon, UK). On Day 1 and Day 2, the medium was replaced with D1–D2 media consisting of 1× NEAA (Life Technologies, #11140035), 1× N2 (Thermofisher, #17502-048), 1× penicillin/streptomycin (Sigma, #P4333), and 55 μM 2-mercaptoethanol (Gibco, #21985023) in DMEM/F-12, GlutaMAX (Gibco, #10565-018) supplemented with 1 μg·mL^−1^ doxycycline (Sigma, #D9891). On Day 3, the medium was replaced with D3 media (1× B27 (Gibco, #12587-010), 1× GlutaMAX (Thermofisher, #35050061), 55 μM 2-mercaptoethanol, 1× penicillin/streptomycin in Neurobasal media (Gibco, #21103-049) supplemented with 1 μg·mL^−1^ doxycycline, 10 ng·mL^−1^ NT3 (Peprotech, #450-03) and 10 ng·mL^−1^ BDNF (Peprotech, #450-02)). On Day 4, cells were detached with Accutase and plated at 60 000 cells per cm^2^ in a poly-D-lysine-coated plate (Sigma, # P1524) in D3 medium supplemented with 1 μg·mL^−1^ doxycycline, 10 ng·mL^−1^ NT3, 10 ng·mL^−1^ BDNF and 10 μM ROCK inhibitor Y-27632. Media was changed on Day 5 and Day 6 without ROCK inhibitor. On Day 8, half of the medium was replaced with D3 media supplemented with 10 ng·mL^−1^ NT3 and 10 ng·mL^−1^ BDNF. Experiments were performed on day 10. Mycoplasma testing was performed regularly, and all experiments were performed with mycoplasma-free cells.

### 2.6 Cerebral organoid cultures and tissue processing

H9 human embryonic stem cells (hESC) (WiCell, WA09, RRID:CVCL_9773, Madison, WI, USA) were cultured at 37 °C on vitronectin-coated plates in TeSR Medium (Stem Cell Technologies, #100-0276). Cerebral organoids were generated using STEMdiff Cerebral Organoid Kit (Stem Cell Technologies, #08570) following the manufacturer’s instructions. Briefly, on Day 0, hESC colonies were detached and dissociated into single cell suspension using Accutase (Life Technologies, #A1110501). 9000 cells were seeded in a 96-well round-bottom ultra-low attachment plate (Corning, #7007) in 100 μL Seeding Media at 37 °C. On Days 2 and 4, 100 μL of Organoid Formation Medium was added to each well. On Day 5, organoids should reach a diameter of 400–600 μm. The nicest round and smooth edges of organoids were selected and transferred into a 6-well ultra-low attachment plate (Appleton Woods, #3471 CC227, Birmingham, UK) containing Induction Media and incubated for 48 h at 37 °C 5% CO_2_ in a humidified incubator. On Day 7, each organoid was embedded in Matrigel (Corning, #354234) for 30 min and placed in Expansion Medium for 3 days. On Day 10, the medium was replaced with Maturation Media and maintained in culture on an orbital shaker at 37 °C 5% CO_2_ in a humidified incubator. The medium was replaced twice a week with Maturation Media and maintained for another 20 weeks. After 2 days of daily treatment with 7 μM Adaptaquin, organoids were fixed for 1 h at room temperature (RT) in 4% paraformaldehyde (PFA) (Sigma, #158127), washed with PBS, and added to 30% PBS-sucrose (Sigma, #S0389) overnight at 4 °C. Organoids were then embedded in OCT (Thermofisher, #15212776) and stored at −80 °C before cryosectioning at 16 μm thickness. Before immuno-histochemistry (IHC), organoid cryosections were washed with PBS, and antigen retrieval was performed by incubating the slides at 80 °C for 10 min in Citrate buffer pH 6 (Sigma, #C9999). Mycoplasma testing was performed regularly, and all experiments were performed with mycoplasma-free cells.

### 2.7 Drug treatments

The following compounds were used in cell cultures: Adaptaquin (AQ) (ApexBio, #C4377, Houston, TX, USA) reconstituted in DMSO (Sigma, #D2438), deferoxamine (DFO) (Sigma, #D9533) reconstituted in water, ammonium iron (III) sulphate dodecahydrate (Ferric iron) (Sigma, #F3629) reconstituted in GSC media, Iron (II) sulphate heptahydrate (Ferrous iron) (Sigma, #F8633) reconstituted in GSC media, cholesterol (Sigma, #C2045) reconstituted in 100% Ethanol, Hemin (Sigma, #H9039) reconstituted in 40 mM NaOH, Zn(II) Mesoporphyrin IX (ZnMP) (Insight Biotechnology, #sc-396862, Welwyn Garden City, UK) reconstituted in DMSO, Mn(III) Protoporphyrin IX chloride (MnPP) (Santa Cruz Biotechnology, #sc-396877A, Heidelberg, Germany) reconstituted in DMSO and Gw3965 (Sigma, #G6295) reconstituted in DMSO. DMSO, water, 40 mM NaOH or 100% EtOH were used as vehicle treatment.

### 2.8 Cytotoxicity measurement

GSCs, DIPGs, primary mouse neural stem cells, primary rat oligodendrocyte precursor cells, primary rat oligodendrocytes, primary rat hippocampal neurons and iPSC-derived neurons were grown in 96-well plates for viability assay. Cell viability was measured by MTT (3-(4,5-dimethylthiazolyl-2)-2,5-diphenyltetrazolium bromide) using the MTT assay kit (Abcam, #ab21109, Cambridge, UK) following the manufacturer’s instructions. Cytotoxicity was measured in a microplate absorbance reader (SPECTROstar Nano, GER). Measurement of cell density was performed in the Incucyte S3 Live-cell Analysis System (Sartorius, Gottingen, Germany) in a humidified incubator at 37 °C, 5% CO_2_. Incucyte images were analysed using INCUCYTE S3 Software v. 2018A.

### 2.9 Iron and cholesterol content measurement

Cells were washed three times with PBS and collected with Accutase (Life Technologies, #A1110501) followed by centrifugation (300 ***g***, 5 min, RT). Cells were lysed in 100 μL RIPA buffer (Sigma, #R0278) on ice followed by incubation at 95 °C for 5 min. The protein concentration of each condition was measured using the Pierce BCA Protein Assay kit (Thermo scientific, #23227) following the manufacturer’s instructions, and absorbance was measured in a microplate reader (SPECTROstar Nano, GER). The remaining cell lysate was used to measure cholesterol content or iron content. Iron concentration was measured using the Iron Assay kit (Sigma, #MAK472) following the manufacturer’s instructions to allow colorimetric detection of total iron in a microplate absorbance reader. Cholesterol concentration was measured using the Amplex Red Cholesterol Assay kit (Thermofisher, #A12216) following the manufacturer’s instructions to allow fluorometric detection of total cholesterol in a microplate fluorescent reader (SpectraMax Spectrofluorometers). The cholesterol or iron content for each condition was defined per milligram of protein.

### 2.10 Immuno-fluorescence

Cells were fixed with 4% paraformaldehyde (Sigma, #441244) for 15 min and washed three times with PBS. Fixed cells were incubated for 1 h in blocking buffer consisting of 3% Bovine Foetal Albumin (Sigma, #A4919), 0.3% Triton X100 (Sigma, #T8787) in PBS. Following incubation, fixed cells were incubated overnight at 4 °C with primary antibodies diluted in blocking buffer: antiOlig2 (RND System, #BAF2418, RRID:AB_2251803, Abingdon, UK) 1 : 200, anti-Pax6 (RND System, #AF8150, RRID:AB_2827378), anti-TFRC (Abcam, #ab84036, RRID:AB_10673794) 1 : 200, anti-MBP (Biorad, #MCA409S, RRID:AB_325004, Watford, UK) 1 : 100, anti-GFAP (Dako, #Z0334, RRID:AB_10013382, Santa Clara, CA, USA) 1 : 400, anti-NFM (Millipore, #AB1987, RRID:AB_91201, Gillingham, UK) 1 : 200, anti-TUJ1 (Abcam, #ab41489, RRID:AB_727049) 1 : 200, anti-mitochondria (Abcam, #ab92824, RRID:AB_10562769) 1 : 200. The next day, cells were washed three times with blocking buffer and incubated for 1 h at room temperature with secondary Alexa Fluor antibodies (Thermofisher) diluted at 1 : 500 in blocking buffer. Following incubation, cells were washed 3 times with blocking buffer, incubated for 15 min with Hoechst (Life Technologies, #H3570, RRID:AB_3675235) diluted at 1 : 2000 in PBS, and then washed once with PBS. Fluorescent images were taken using the Operetta CLS High-Content Analysis System (PerkinElmer, Shelton, CT, USA) and analysed in HARMONY v. 4.9 and Omero (Open Microscopy Environment).

### 2.11 Reactive oxygen species, Calcein-AM, ZnMP imaging

Reactive oxygen species was measured using CellROX Green Reagent (Thermofisher, #C10448) following the manufacturer’s instructions. Briefly, cells were treated with 5 μM CellROX reagent in GSC media for 30 min in a humidified incubator at 37 °C, 5% CO_2_. Following incubation, cells were washed twice with PBS and imaged directly using the EVOS M5000 Imaging System (Thermofisher). Images were analysed using FIJI [28] (RRID:SCR_003070) and CellROX signal was quantified using CELL PROFILER v.4.2 [29].

Living cells were imaged using the LIVE/DEAD Viability/Cytotoxicity kit (Sigma, #L3224).

Living cells were labelled with Calcein-AM, a cell-permeant dye that is converted by intracellular esterases into a green fluorescent form, allowing the visualization of viable cells. Cells were treated with 0.5 μM Calcein-AM and incubated for 20 min in a humidified incubator at 37 °C, 5% CO_2_. Following incubation, cells were washed twice with PBS and imaged directly using the EVOS M5000 Imaging System.

Uptake of Zn(II) Mesoporphyrin IX (ZnMP) (Insight Biotechnology, #sc-396862) was measured by treating the cells with 1, 5, or 10 μM of ZnMP for 30 min in a humidified incubator at 37 °C, 5% CO_2_. Following incubation, cells were fixed for 15 min with 4% Paraformaldehyde (Sigma, #441244), washed twice with PBS and incubated for 15 min with DAPI (Life Technologies, #H3570, RRID:AB_3675235). Images were acquired in a DMi8 Inverted confocal microscope (Leica, Milton Keynes, UK). Images were analysed using FIJI [30] (RRID:SCR_003070) and the ZnMP signal was quantified using CELL PROFILER v.4.2 [31].

### 2.12 Western blot

Cells were washed with ice-cold PBS and lysed with ice-cold 2× SDS sample buffer supplemented with denarase (c-LEcta, #20804-100k, Leipzig, Germany) (20 mL of 6× SDS sample buffer contain 6 mL glycerol (Sigma, #G6279), 3 mL 1 M Tris pH 6.8 (Sigma, #678937), 1.2 g SDS (Sigma, #L3771), 930 mg DTT (Sigma, #D0632), 2 mg bromophenol blue (Sigma, #B0126) in water). Cells were collected in precold tubes using a cell scraper (Fisher Scientific, #08100241) and immediately stored at −80 °C. Defrosted samples were incubated at 95 °C for 5 min and centrifuged for 5 min at 12 000 ***g***. 10 μL of the supernatant was loaded on 4–12% Bis Tris NuPAGE gels (Thermofisher, #NP0335BOX) according to the manufacturer’s instruction and protein migration was done with 90 volts for 15 min followed by 120 volts. After migration, the proteins were transferred to PVDF membranes (Millipore, #IPFL00010) using ice-cold Bolt Transfer Buffer (Thermofisher, #BT00061) for 1 h with 15 volts. Membranes were blocked in blocking buffer consisting of 3% nonfat milk bovine (Sigma, #M7409) in TBSt (1× TBS buffer (Sigma, #T5912)) supplemented with 0.1% Tween20 (Sigma, #93773) for 1 h. Following incubation, membranes were incubated overnight at 4% with gentle agitation with primary antibodies diluted at 1/1000 in blocking buffer: anti-TFRC (Abcam, #ab84038), anti-FTH1 (Mybiosource, #MBS9606937, San Diego, CA, USA), anti-FTL (Atlas, # HPA041602), anti-HMGCS1 (Proteintech, # 17643-1-AP), anti-SQLE (Proteintech, #12544-1-AP), anti-HIF1α (Cell Signalling, #36169T, RRID: AB_2799095, Leiden, Netherlands), anti-PHD2 (Novus Biologicals, #NB100137, RRID:AB_3149061, Abingdon, UK), anti-MTCO1 (Abcam, #ab14705, RRID: AB_2084810), anti-Cytochrome C (Abcam, #ab110325, RRID:AB_10864775). The next day, membranes were washed three times for 5 min with TBSt and incubated for 1 h at room temperature with Li-COR secondary antibodies diluted in blocking buffer (1/5000). Membranes were washed again three times for 5 min with TBSt and proteins were detected using Li-COR Odyssey (LI-COR Biosciences, Cambridge, UK). Images were analysed using FIJI [30] (RRID:SCR_003070).

### 2.13 RNA isolation, cDNA synthesis and RT-qPCR

Cells were washed with ice-cold PBS and lysed in TriReagent (Sigma, #678937). mRNA was purified using Direct-zol RNA micro prep (Zymo Research, #R2062, Freiburg, Germany) following the manufacturer’s instructions, and concentration was measured using Nanodrop (Thermofisher, Waltham, MA, USA). cDNA was synthesized using the ZymoScript RT Pre-Mix kit (Zymo Research, #R3012) following the manufacturer’s instructions. Quantitative real-time PCR (RT-qPCR) was conducted using the QUANTSTUDIO 12K Flex Real-Time PCR System (Applied Biosystems). The reactions were carried out with PowerUp SYBR Green Master Mix (Applied Biosystems, #A25741, Waltham, MA, USA) following the manufacturer’s guidelines. Gene expression levels were standardized against β-actin expression.

Primers used:

**Table:** 

Gene name	Forward primer	Reverse primer
TRIB3 (h)	GCGTGATCTCAAGCTGTGT	GCTTGTCCCACAGGGAATCA
CHOP (h)	GTTCCAGCCACTCCCCATTA	GTGTCCCGAAGGAGAAAGGC
ATF4 (h)	TCAGTCCCTCCAACAACAGC	GGCATCCAAGTCGAACTCCT
TFRC (h)	CTGGCTCGGCAAGTAGATGG	TGTGACATTGGCCTTTGTGT
FTH1 (h)	TGACCCCCATTTGTGTGACT	TCACGTGGTCACCCAATTCTT
FTL (h)	AGCGTCTCCTGAAGATGCAA	TTTCATGGCGTCTGGGGTTT
Actin (h)	TTCGCGGGCGACGAT	GGAATCCTTCTGACCCATGCC
HMGCS1 (h)	ATGCATGCTATGGAGGCACA	TACCAGGGCATACCGTCCAT
SQLE (h)	GCCTGCCTTTCATTGGCTTC	TTCCTTTTCTGCGCCTCCTG
MSMO1 (h)	AGCATCCTTGGCTGTGGAAT	CATGTTGCAATCTGGAACTTTGT
INSIG1 (h)	ACCACGCCAGTGCTAAATTG	ATGTCCACCAAAGGCCCAAA
PHD2 (h)	CGTCGCAACCCTCATGAAGTA	TCGTGCTCTCTCATCTGCATC
PHD1 (h)	CTTCGTCAGAGCCCTTGGAG	TCCTGGACAGTGGTAGGAGG
PHD3 (h)	GCTGGACCTGGAGAAAATTGC	CACCTCGCCCAGGAAGTTGT
HIF1a (h)	GCAGAATGCTCAGAGAAAGCG	TAGCTGCATGATCGTCTGGC
HIF1b (h)	GACTACTGCCAACCCCGAAA	ACAATGGCTCCTCCACCTTG
HIF2a (h)	ATGACAGCTGACAAGGAGAAGAA	TGGGCCAGCTCATAGAACAC

### 2.14 RNA sequencing

Cells were washed with ice-cold PBS and lysed in TriReagent (Sigma, #678937). mRNA was purified using Direct-zol RNA micro prep (Zymo Research, #R2062) following the manufacturer’s instructions. mRNA concentration and integrity were measured using the RNA ScreenTape system (reagent: Agilent, #5067-5577, RNA SceenTape: Agilent, 5067-5576, TapeStation: 4200 TapeStation System, Agilent). High-quality mRNA was subjected to library preparation by QIAseq UPXome RNA Library (polyA) (Qiagen, Manchester, UK) and bulk sequencing in NovaSeqX_25B_UpTo300 (Illumina, San Diego, CA, USA). Raw paired-end FASTQ files underwent initial quality assessment with FASTQC. Illumina adapter sequences and low-quality bases were then trimmed using TRIMMOMATIC (v0.39) (RRID:SCR_011848), ensuring that only high-quality reads were retained for downstream analysis. The cleaned reads were aligned to the human reference genome (GRCh38/hg38) using the STAR aligner (v2.7.10a) within a Singularity container. STAR was run with default parameters and the --quantMode TranscriptomeSAM option enabled, which produces transcriptome-aligned reads for downstream quantification. Gene- and transcript-level expression was quantified using RSEM (v1.3.3). RSEM was executed in paired-end mode with the STAR transcriptome-aligned reads as input, generating both raw read counts and normalized expression values (transcripts per million, TPM). Quality control reports from all steps (FASTQC, TRIMMOMATIC (RRID: SCR_011848), STAR, and RSEM) were aggregated using

MultiQC to provide a comprehensive overview of data quality. Finally, downstream analyses—including differential expression testing, pathway enrichment analysis, unsupervised clustering, and data visualization—were performed using the OmicsPlayground platform (v2.8.19) with an FDR = 0.05. Heatmaps were generated using Heatmapper (). MCL clustering (inflation parameter = 1.5) and DBSCAN clustering (epsilon = 15) were generated using STRING V.12.0 (www.string-db.org) (RRID:SCR_005223).

### 2.15 Preparation of cell pellets for MRI and fluorescence imaging

GSCs and HEK293T (ATCC, CRL-3216, RRID: CVCL_0063) cells were cultured in flasks until they reached 80% confluency. Cells were then treated with 0.9 mM Mn(III) Protoporphyrin IX chloride (MnPP) (Santa Cruz Biotechnology, #sc-396877A) in 9% DMSO and 91% GSC media, 10 μM Zn(II) Mesoporphyrin IX (ZnMP) (Insight Biotechnology, #sc-396862) in 9% DMSO and 91% GSC media, or 9% DMSO (as a negative control) and 91% GSC media and incubated for 1 h in a humidified incubator at 37 °C with 5% CO. Following incubation, cells were detached using Accutase (Life Technologies, #A1110501) and centrifuged at 300 ***g*** for 5 min. The resulting cell pellet was resuspended in 10 mL PBS for washing. A total of four washes were performed before the cells were counted and resuspended in 100 μL of PBS. The cell suspensions were then transferred into 300-μL microcentrifuge tubes, and the final cell pellet was obtained by centrifugation. Pellets were immediately placed on ice and imaged on the same day.

### 2.16 Near infra-red fluorescence imaging of cell pellets

Cell pellets were imaged using the Pearl Trilogy Small Animal Imaging System (LI-COR Biosciences, Lincoln, NE, USA) at an excitation wavelength of 685 nm and detection at 820 nm as well as White Light at a resolution of 170 μm. Images were analysed using IMAGE STUDIO software (LI-COR Biosciences) with manual ROIs.

### 2.17 Magnetic resonance imaging (MRI)

Experiments were performed at 7 Tesla (Agilent, Palo Alto, CA, USA) using a 40-mm-diameter Millipede quadrature volume coil for ^1^H transmit and receive (Agilent). To determine the water proton *T*_1_ in the pellets, an inversion recovery fast low angle shot (FLASH) sequence (Agilent), which was modified to include a non-slice-selective adiabatic inversion pulse for rapid *T*_1_ mapping of a small number of slices, was used with the following inversion times: 0.05, 0.1, 0.2, 0.5, 1, 2, 3 and 4 s. *T*_1_ was determined using the VNMRJ 3.1.A software (Agilent). The relaxation rate *R*_1_ was calculated by taking the inverse of *T*_1_ for every voxel using MATLAB (Mathworks, Natick, MA, USA) (RRID:SCR_001622). By means of the formula below and the previously determined relaxivity *r*_1_ of Mn-PP, the contrast agent concentration in the pellet was deduced.

$${\left[\text{C}\right]}_{\text{Mn}-\text{PP}}=\frac{\frac{1}{T_{1\_\text{obs}\;}}-\frac{1}{T_{1_{t}}}}{r_{1}}$$

where [C]_Mn-PP_ is the concentration of Mn-PP in the cell pellet, *T*_1_obs_ the *T*_1_ observed in the pellet, $T_{1_{t}}$ the *T*_1_ in the corresponding control pellet and *r*_1_ the relaxivity of Mn-PP at the same field strength. The images of the *T*_1_ and *R*_1_ maps were generated using MATLAB (Mathworks) (RRID:SCR_001622), whereas the *T*_2_-weighted and inversion recovery images were generated using FIJI [28] (RRID:SCR_003070).

### 2.18 Gene silencing

GSCs were transfected with siRNA targeting PHD2 (Santa Cruz Biotechnology, #sc-45537), HIF1a (Santa Cruz Biotechnology, #sc-35561) or control (Santa Cruz Biotechnology, #sc-37007) using Lipofectamine RNAi-MAX Transfection reagent (Thermofisher, #13778075) following the manufacturer’s instructions. Two days after Lipofectamine transfections, cells were collected for RT-qPCR analysis or western blot analysis to confirm gene and protein knockdown, or treated with Adaptaquin alone or in combination with iron chelators for two days for cytotoxicity analysis.

### 2.19 Lentiviral preparation and stable genetically engineered GSC lines

Lentiviral plasmids for overexpression of PHD2, HIF1a and GFP: pLV[Exp]-Puro-CBh>hEGLN1 [NM_022051.3]/HA, pLV[Exp]-Puro-CBh>hHIF1A [NM_001243084.2]/HA, pLV[Exp]-Puro-CBh>EGFP were constructed by VectoBuilder (Vector ID: VB240910-1340xpn, VB230911-1228zba). HEK293T (ATCC, CRL-3216, RRID:CVCL_0063) were plated on Poly-D-lysin coated plates and cultured with DMEM/F12 (Gibco, #11320033) supplemented with 10% fetal bovine serum (Thermo Scientific, #A5256701). When HEK cells reached 80% confluency, lentivirus was generated by transfecting the cells with second-generation lentiviral packaging plasmid (VSV.G: Addgene, Plasmid #14888, RRID: Addgene_14888 and psPAX2: Addgene, Plasmid #12260, RRID:Addgene_12260, Watertown, MA, USA) using Lipofectamine 2000 Transfection reagent (Thermofisher, #11668027) following the manufacturer’s instructions. The day after transfection, media was replaced with GSC media and cells were incubated for 24–48 h in a humidified incubator at 37 °C, 5% CO_2_. Following incubation, media was collected, and the floating cells were removed by centrifugation at 1000 ***g*** for 10 min. The supernatant was then passed through a 0.45 μM syringe filter to remove cell debris. The supernatant containing lentiviral particles was frozen at −80 °C or used directly on GSCs. GSCs were cultured in Laminin-coated flasks in GSC media. When cells reached 40–50% confluency, media was replaced with 50% fresh GSC media and 50% of the supernatant containing lentiviral particles and incubated for 3 days in a humidified incubator at 37 °C, 5% CO_2_. Following incubation, media was replaced with GSC media supplemented with 1 μg·mL^−1^ puromycin (Fisher Scientific, #11497610). Cells were maintained in GSC media with puromycin for 1–2 weeks, until no floating dead cells or cytotoxicity was observable in the culture. RT-qPCR analysis and western blotting analysis were performed on the genetically engineered GSCs to confirm the overexpression of PHD2 or HIF1a. Cells were then stored in liquid nitrogen in GSC media containing 10% DMSO.

### 2.20 Illustrations

All artwork was created using Adobe Illustrator CC 21.1.0 (Adobe Systems, San Jose, CA, USA) (RRID: SCR_010279), POWERPOINT V 16.94 (Microsoft, Redmond, WA, USA) or BioRender (www.biorender.com) under the terms of an academic license.

### 2.21 Statistical analyses

All data are represented as mean ± SEM. Significance using the two-tailed unpaired Student’s *t*-test and/or one-way ANOVA was performed using GRAPHPAD software (Boston, MA, USA) (RRID:SCR_002798). *, *P* < 0.05; **, *P* < 0.01; ***, *P* < 0.001; ns, not significant. RNA sequencing analysis was analysed using a false discovery rate (FDR) of 0.05.

## 3 Results

### 3.1 Adaptaquin is selectively cytotoxic to glioma stem cells

**Fig. 1:**
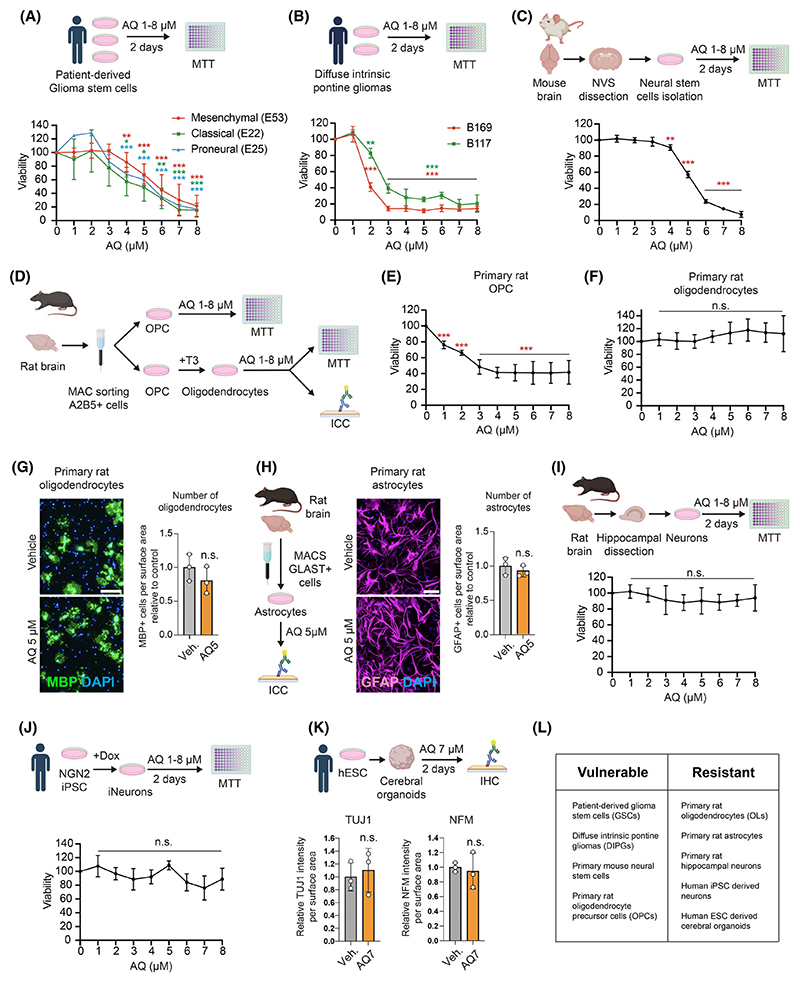
Adaptaquin is selectively cytotoxic to glioma stem cells. (A, B) Viability of patient-derived glioma stem cell lines (A) and diffuse intrinsic pontine glioma cell lines (B) treated with Adaptaquin for 2 days, measured by MTT (3-(4,5-dimethylthiazol-2-yl)-2,5 diphenyl tetrazolium bromide) assay (*n* = 3–9). (C) Viability of primary mouse neural stem cells treated with Adaptaquin for 2 days, measured by MTT assay (*n* = 3). (D) Schematic representation of oligodendrocyte precursor cell isolation from rat brains and oligodendrocyte maturation. (E, F) Viability of primary rat oligodendrocyte precursor cells (E) and mature oligodendrocytes (F) treated with Adaptaquin for 2 days, measured by MTT assay (*n* = 5). (G) Immunofluorescence imaging of the oligodendrocyte marker myelin basic protein (MBP) together with DAPI of primary rat oligodendrocyte cultures treated with 5 μM Adaptaquin for 2 days, with quantification (*n* = 3). (H) Immunofluorescence imaging of the astrocyte marker glial fibrillary acidic protein (GFAP) together with DAPI of primary rat astrocyte cultures treated with 5 μM Adaptaquin for 2 days, with quantification (*n* = 3). (I, J) Viability of primary rat hippocampal neurons (I) and human iPSC-derived neurons (J) treated with Adaptaquin for 2 days, measured by MTT assay (*n* = 3–4). (K) Quantification of the neuronal markers Neurofilament M (NFM) and Beta-3 tubulin (TUJ1) together with DAPI of 10-week-old hESC-derived cerebral organoids after 2 days of treatment with 7 μM Adaptaquin (*n* = 3). (L) Summary table of vulnerable and resistant cell types to Adaptaquin treatment. Data are represented as mean ± SEM. Significance using the two-tailed unpaired Student’s *t*-test for G, H, K and one-way ANOVA for A, B, C, E, F, I, J. *, *P* < 0.05; **, *P* < 0.01; ***, *P* < 0.001; ns, not significant. Scale bar: 100 μm.

To explore the ability of AQ to induce GSCs death, we treated patient-derived GSC lines (WHO grade IV, IDH wild-type) classified as classical (E22), proneural (E25) or mesenchymal (E53), with several concentrations of AQ for 2 days. As shown (Fig. 1A), AQ (up to 4 μM) reduced the viability of all GSCs subtypes. AQ treatment of diffuse intrinsic pontine glioma lines (DIPG) also strongly reduced viability (Fig. 1B). We next assessed the potential toxicity of AQ in noncancerous cells. While AQ shows strong toxicity towards proliferative primary mouse neural stem cells (NSCs) and oligodendrocyte precursor cells (OPCs) (Fig. 1C–E), primary rat oligodendrocytes, astrocytes and hippocampal neurons were unaffected (Fig. 1F–I). We next investigated AQ effects on human induced pluripotent stem cells (iPSC)-derived neurons and human embryonic stem cells (hESC)-derived cerebral organoids. Human iPSC-derived neurons treated with increasing concentrations of AQ for 2 days showed no reduction in viability (Fig. 1J). Similarly, 20-week-old hESC-derived cerebral organoids exposed to a high dose of AQ (7 μM) for 2 days retained normal immuno-reactivity for neuronal markers TUJ1 and neurofilament-M (NFM) (Fig. 1K and Fig. S1). In summary, these findings demonstrate that AQ selectively targets proliferative GSCs and precursor cells, such as OPCs and NSCs, while being nontoxic to human neurons and nonproliferative brain cells (Fig. 1L).

### 3.2 Transcriptomic dysregulation of iron transport and cholesterol metabolism by AQ

**Fig. 2:**
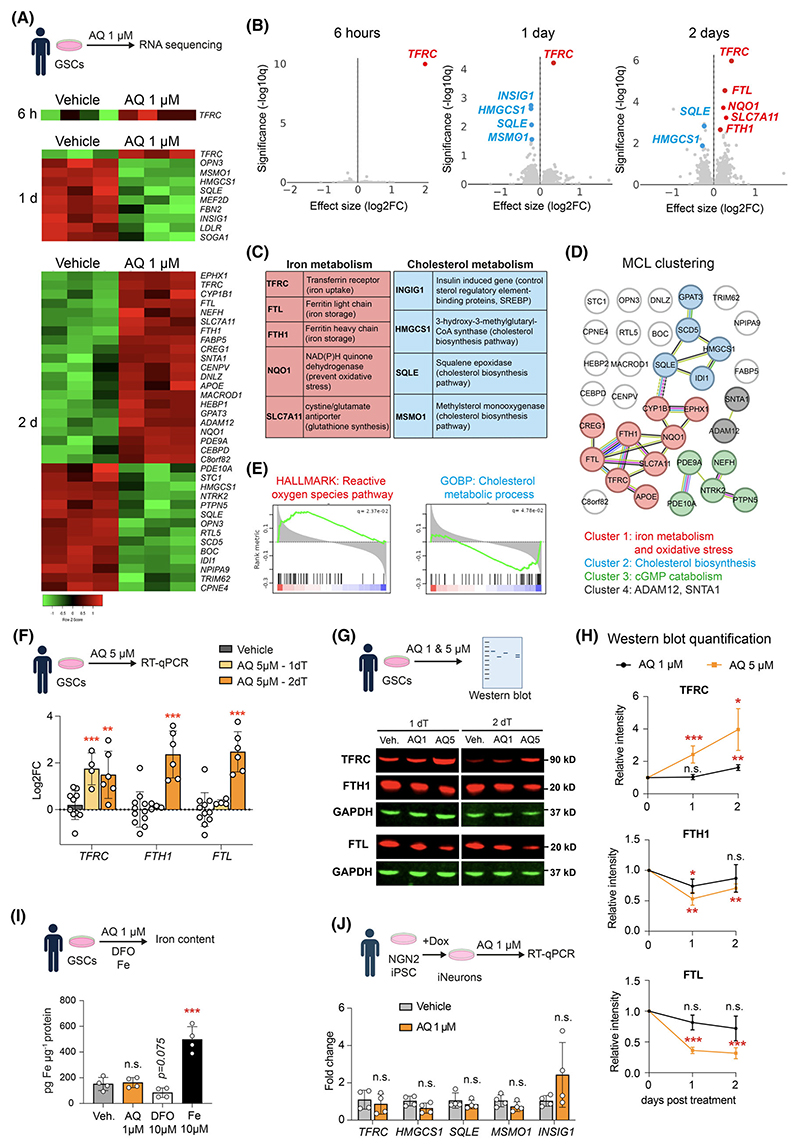
Transcriptomic dysregulation of iron transport and cholesterol metabolism by AQ. (A) Heatmap representation of differentially expressed genes (DEG) in glioma stem cells (GSCs) after 6 h, 1 day or 2 days treatment with 1 μM Adaptaquin (AQ). (B) Volcano plot representation showing DEGs after 6 h, 1 day and 2 days of treatment with 1 μM Adaptaquin compared to vehicle-treated GSCs, highlighting upregulated iron-related genes in red and cholesterol-related genes in blue. (C) List of the main upregulated genes involved in iron metabolism (red) and downregulated genes involved in cholesterol metabolism (blue), along with their protein functions. (D) Markov cluster algorithm (MCL) clustering of DEGs altered after 2 days treatment with 1 μM AQ. (E) Plot showing the top enriched gene sets after 2 days treatments with 1 μM AQ. (F) Quantification of Transferrin receptor (TFRC), Ferritin heavy chain (FTH1), Ferritin light chain (FTL) expression level in GSCs treated with 5 μM AQ for 1 or 2 days, measured by RT-qPCR relative to actin (*n* = 4–9). (G, H) Western Blot analysis of TFRC, FTH1, FTL in GSCs treated with 1 or 5 μM AQ for 1 or 2 days (G), with quantification (H) relative to GAPDH (*n* = 3). (I) Measurement of iron content in GSCs treated for 2 days with vehicle, AQ, Deferoxamine or ferric iron (*n* = 4). (J) Quantification of TFRC, squalene epoxidase (SQLE), 3-hydroxy-3-methylglutaryl-CoA synthase (HMGCS1), methylsterol monooxygenase (MSMO1), insulin induced gene (INSIG1) expression levels in iPSC-derived neurons after 1 day of treatment with 1 μM AQ, measured by RT-qPCR relative to actin (*n* = 4). Data are represented as mean ± SEM. Significance using the two-tailed unpaired Student’s *t*-test for F, H, I, J. *, *P* < 0.05; **, *P* < 0.01; ***, *P* < 0.001; ns, not significant.

Previous studies have demonstrated that AQ exhibits a neuroprotective effect at a concentration of 1 μM [23,24]. Given that the central concept of our study is to explore the potential repurposing of a neuroprotective compound as an anticancer agent, we evaluated whether this concentration could also induce transcriptional changes in GSCs. Therefore, we conducted bulk RNA sequencing of mesenchymal-like line E53. Our finding revealed transcriptional dysregulation of 1, 10 and 35 differentially expressed genes (DEG) significantly altered after 6 h, 1 day and 2 days of treatment, respectively (Fig. 2A). Notably, the top DEG was Transferrin receptor C (TFRC), upregulated as early as 6 h and maintained for a further 2 days (Fig. 2A,B). In addition, genes involved in iron metabolism, such as *FTL* and *FTH1*, were also upregulated after 2 days of treatment (Fig. 2A–C). Interestingly, we also observed an upregulation of the ROS responsive pathways, suggesting that AQ treatment may contribute to elevated oxidative stress (Fig. 2D,E). To assess the dose dependence of this response, we evaluated iron metabolism gene expression following treatment with a higher, cytotoxic concentration of AQ (5 μM) for 24 and 48 h by RT-qPCR. Consistent with the 1 μM treatment, *TFRC* was upregulated after 1 day, and *TFRC, FTL*, and *FTH1* were all upregulated after 2 days (Fig. 2F). However, protein expression analysis revealed a divergence from mRNA trends. While TFRC protein levels increased at both 1 and 5 μM AQ, FTL and FTH1 protein levels were downregulated, indicating a post-transcriptional decoupling (Fig. 2G, H). Interestingly, despite these transcriptional and translational changes, the cellular iron pool was unchanged after AQ treatment as compared to ferrous iron treatment, which increased the iron pool (Fig. 2I), consistent with previous findings indicating that AQ is not an iron chelator [23]. Among the most significantly downregulated DEG were those for cholesterol metabolism genes, including *INSIG1, HMGCS1, SQLE* and *MSMO1* (Fig. 2A–C). Gene ontology analysis and MLC clustering of proteins encoding DEGs altered after 2 days of treatment with 1 μM AQ confirmed downregulation of cholesterol biosynthetic and metabolic processes (Fig. 2D,E). In contrast, human neurons treated with 1 μM AQ for 2 days did not exhibit regulation of *TFRC* nor cholesterol metabolic genes *INSIG1, HMGCS1, SQLE* and *MSMO1* (Fig. 2J).

### 3.3 AQ toxicity is potentiated by iron chelation

**Fig. 3:**
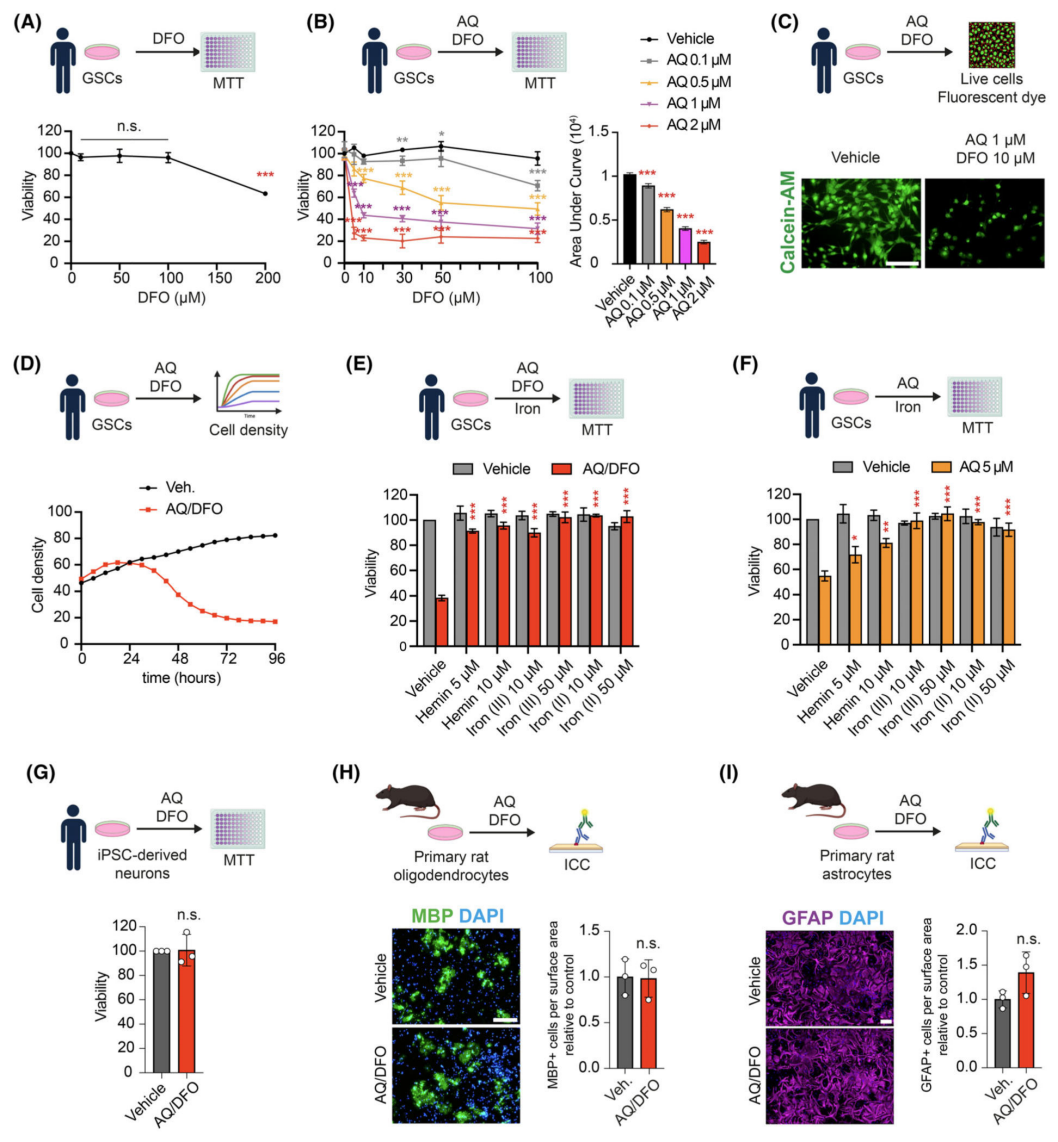
Adaptaquin toxicity is potentiated by iron chelation. (A) Viability of glioma stem cells (GSCs) treated with Deferoxamine (DFO) for 2 days, measured by MTT (3-(4,5-dimethylthiazol-2-yl)-2,5 diphenyl tetrazolium bromide) assay (*n* = 4). (B) Viability of GSCs treated with DFO and Adaptaquin (AQ) for 2 days, measured by MTT assay (*n* = 4–8), with measure of Area Under Curve (AUC). (C) Immunofluorescent imaging of Calcein-AM in GSCs treated with the combination Adaptaquin 1 μM/deferoxamine 10 μM (AQ/DFO) or vehicle for 2 days. (D) Measure of GSCs cell density treated with AQ/DFO over 4 days (*n* = 4). (E, F) Viability of GSCs treated with Hemin, ferric iron (Iron (III)) and ferrous iron (Iron (II)) in combination with AQ/DFO (E) or 5 μM AQ (F) for 2 days, measured by MTT assay (*n* = 3). (G) Viability of iPSC-derived neurons treated with AQ/DFO for 2 days, measured by MTT assay (*n* = 3). (H) Immunofluorescence imaging of the oligodendrocyte marker myelin basic protein (MBP) together with DAPI of primary rat oligodendrocyte cultures treated with vehicle or AQ/DFO for 2 days, with quantification (*n* = 3). (I) Immunofluorescence imaging of the astrocyte marker glial fibrillary acidic protein (GFAP) together with DAPI of primary rat astrocyte cultures treated with vehicle or AQ/DFO for 2 days, with quantification (*n* = 3). Data are represented as mean ± SEM. Significance using the two-tailed unpaired Student’s *t*-test for E, F, G, H, I and one-way ANOVA for A, B. *, *P* < 0.05; **, *P* < 0.01; ***, *P* < 0.001; ns, not significant. Scale bar: 100 μm.

Given findings of upregulated *TFRC*, we investigated whether iron availability plays a role in AQ-mediated GSC death. While iron chelators such as Deferoxamine (DFO) alone reduced GSC viability at high concentration (up to 200 μM) (Fig. 3A), we found that cotreatment of GSCs with AQ and DFO greatly enhanced the toxicity of low-dose AQ (Fig. 3B). Specifically, a combination of 1 μM AQ and 10 μM DFO (AQ/DFO) reduced GSC viability by more than 50% (Fig. 3B–D). Consistently, the addition of heme-iron, ferric iron or ferrous iron mitigated AQ toxicity, both alone and in combination with iron chelators (Fig. 3E, F). These findings indicate that AQ-induced dysregulation of iron metabolism contributes to GSC death. In contrast, human neurons did not show any susceptibility to the combined treatment of AQ 1 μM/DFO 10 μM (Fig. 3G). Similarly, primary rat oligodendrocytes and astrocytes were not affected by these treatments (Fig. 3H,I). These findings indicated that AQ can selectively dysregulate iron metabolism in highly proliferative cells such as GSCs, but not neurons, and that its toxicity is potentiated by iron chelators.

### 3.4 Uptake of heme analogues and functional heme transport in GSCs

Results above indicate that AQ sensitizes GSCs to iron deprivation. Interestingly, the addition of iron, especially in the form of heme, reduces AQ toxicity, suggesting hemin can act as an iron source (Fig. S2A). While GSCs have been reported to exhibit dysregulated heme biosynthesis [32,33], no heme transporter has been identified in GSCs. Therefore, we investigated a potential GSC heme transporter by testing cellular uptake of various heme analogues (Fig. S2B). Iron is released from hemin through oxidative degradation catalysed by heme oxygenases (HMOX1 and HMOX2), and this reaction is inhibited by zinc mesoporphyrin (ZnMP), a competitive heme analogue (Fig. S2C). Interestingly, we found that the protective effect of hemin against AQ/DFO-induced cell death was antagonized by ZnMP (Fig. S2D), suggesting that hemin’s protective role is dependent on iron release. This finding highlights the functional relevance of heme-derived iron in mitigating AQ/DFO toxicity and suggests an alternative pathway for Fe import via heme. Further supporting this interpretation, we show that hemin supplementation protects GSCs from DFO-induced cell death (Fig. S2E).

To determine whether iPSC-derived neurons also utilize hemin to mitigate DFO toxicity, we conducted similar experiments. Interestingly, while neurons appeared less susceptible to iron deprivation, hemin failed to rescue them from DFO toxicity (Fig. S2F). Second, we took advantage of the fluorescent properties of the heme analogue Zinc Mesoporphyrin (ZnMP). Fluorescence analysis revealed that GSCs treated with increasing doses of ZnMP exhibited a stronger fluorescent signal (Fig. S2G). Additionally, fluorescence measurements of the cell pellet confirmed enhanced ZnMP uptake in ZnMP-treated GSCs compared to ZnMP-treated HEK or DMSO-treated controls (Fig. S2H). We also examined the uptake of Manganese Mesoporphyrin (MnPP), a heme analogue detectable via MRI. R_1_ relaxation rate measurements together with the relaxivity of MnPP (Fig. S2I) allowed quantification of MnPP concentration in the cell pellet using MRI. These results together demonstrate that GSCs show a higher uptake of MnPP compared to HEK cells (Fig. S2J), indicating a functional heme transporter in GSCs. Moreover, they indicate that heme can provide an alternative source of iron, a finding of more general significance, considering the interest in using therapies for GBM targeted to iron metabolism.

### 3.5 AQ and AQ/DFO toxicity is independent of PHD2 and HIF1*α*

**Fig. 4:**
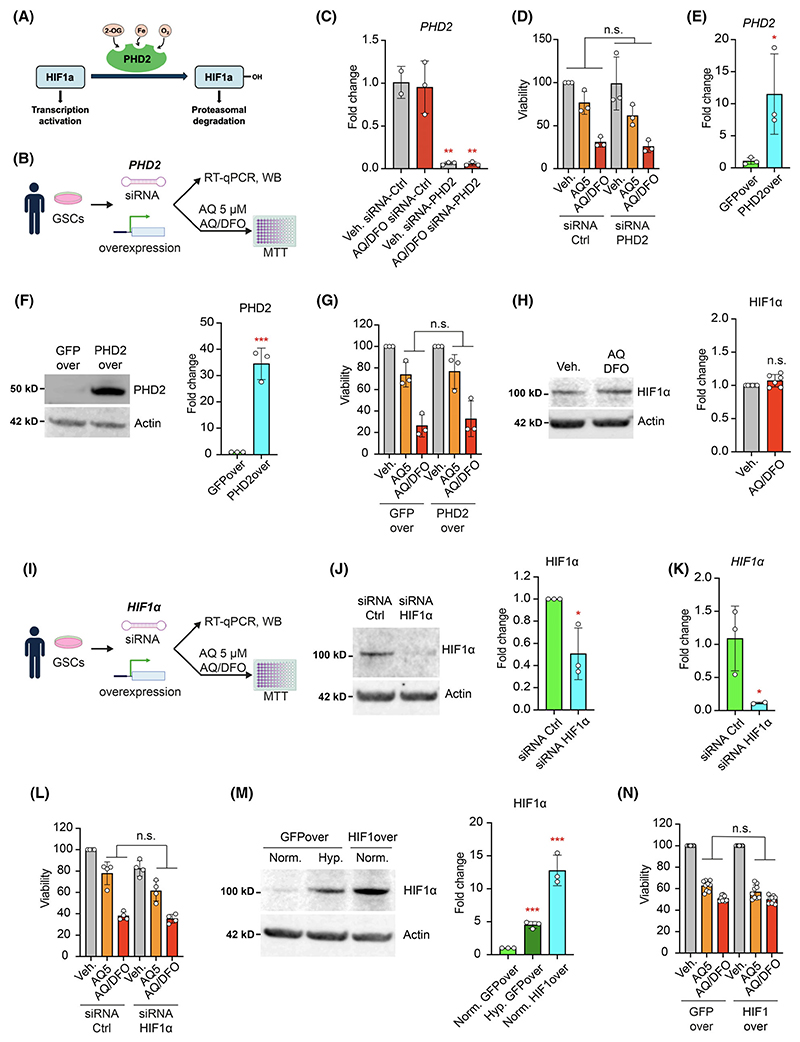
AQ and AQ/DFO toxicity is independent of PHD2 and HIF1α. (A) Schematic representation of hypoxia inducible factor 1α (HIF1α) stability regulation by prolyl hydroxylase 2 (PHD2). (B) The role of PHD2 in Adaptaquin (AQ) and the combination Adaptaquin 1 μM/deferoxamine 10 μM (AQ/DFO)-mediated glioma stem cell (GSC) death was assessed using PHD2 loss-of-function (siRNA) and gain-of-function (lentiviral overexpression). (C) Quantification of *PHD2* expression level in GSCs treated with vehicle or AQ/DFO for 1 day and transfected with siRNA control or siRNA targeting *PHD2*, measured by RT-qPCR relative to actin (*n* = 2–3). (D) Viability of GSCs treated for 2 days with vehicle, 5 μM AQ or with AQ/DFO and transfected with siRNA control or siRNA targeting *PHD2*, measured by MTT (3-[4,5-dimethylthiazol-2-yl]-2,5 diphenyl tetrazolium bromide) assay (*n* = 3). (E) Quantification of *PHD2* expression levels in GSCs overexpressing GFP (GFPover) and PHD2 (PHD2over), measured by RT-qPCR relative to actin (*n* = 3). (F) Western Blot of PHD2 in GSCs GFPover and PHD2over, with quantification relative to actin (*n* = 3). (G) Viability of GSCs GFPover and PHD2over treated with vehicle, 5 μM AQ or with AQ/DFO for 2 days, measured by MTT assay (*n* = 3). (H) Western Blot of HIF1α in GSCs treated with vehicle or with AQ/DFO for 1 day, with quantification relative to actin (*n* = 6). (I) The role of HIF1α in AQ and AQ/DFO-mediated GSC death was assessed using HIF1α loss-of-function (siRNA) and gain-of-function (lentiviral overexpression). (J) Western Blot of HIF1α in GSCs transfected with siRNA control or siRNA targeting *HIF1α*, with quantification relative to actin (*n* = 3). (K) Quantification of *HIF1α* expression level in GSCs transfected with siRNA control or siRNA targeting *HIF1α*, measured by RT-qPCR relative to actin (*n* = 2–3). (L) Viability of GSCs treated with vehicle, 5 μM AQ or with AQ/DFO for 2 days and transfected with siRNA control or siRNA targeting *HIF1α*, measured by MTT assay (*n* = 4). (M) Western Blot of HIF1α in GSCs overexpressing GFP (GFPover) and HIF1α (HIF1over) in normoxic (20% oxygen) and hypoxic (1% oxygen) conditions, with quantification relative to actin (*n* = 3). (N) Viability of GSCs GFPover and HIF1over treated for 2 days with vehicle, 5 μM AQ and with AQ/DFO, measured by MTT assay (*n* = 9). Data are represented as mean ± SEM. Significance using the two-tailed unpaired Student’s *t*-test. *, *P* < 0.05; **, *P* < 0.01; ***, *P* < 0.001; ns, not significant.

AQ has been identified as an inhibitor of HIF-PHD2 [23] (Fig. 4A). To determine whether PHD2 inhibition underlies cell death induced by AQ or AQ/DFO, we targeted *PHD2* in GSCs using small RNA interference (siRNA) (Fig. 4B). Quantitative RT-PCR revealed a significant reduction in *PHD2* transcript both in the vehicle and after 1 day of AQ/DFO treatment compared to control siRNA (Fig. 4C and Fig. S3A,B). Nevertheless, GSCs treated with AQ alone or in combination with DFO exhibited similar levels of toxicity regardless of *PHD2* knockdown compared to control siRNA (Fig. 4D). We next generated a stable GSC line overexpressing PHD2 using lentiviral infection followed by puromycin selection (Fig. 4B,E,F and Fig. S3C,D). Measurement of cell viability after AQ treatment alone or in combination with deferoxamine, however, showed similar toxicity in both the GFP overexpressing line and PHD2 overexpressing line (Fig. 4G). Moreover, despite PHD’s role in stabilizing HIF1α, our results indicate that HIF1α levels in GSCs remained unchanged after AQ/DFO treatment (Fig. 4H). These results suggested that AQ and AQ/DFO toxicity are independent of PHD2 levels.

In addition, we knocked down HIF1α in GSCs by 50% using siRNA (Fig. 4I–K and Fig. S3E,F), but the toxicity of AQ or AQ/DFO was unaffected (Fig. 4L). Similarly, we generated a stable GSC line overexpressing HIF1α using lentiviral infection followed by puromycin selection (Fig. 4M). HIF1α overexpression had no effect on AQ and AQ/DFO susceptibility (Fig. 4N), confirming previous findings in rodent cells showing HIF1α independent effect of AQ [23,25]. Together, these findings indicate that AQ toxicity alone or in combination with iron chelators in GSCs is independent of PHD2 and HIF1α regulation.

### 3.6 The combination of AQ and iron chelators specifically impairs cholesterol metabolism in GSCs

**Fig. 5:**
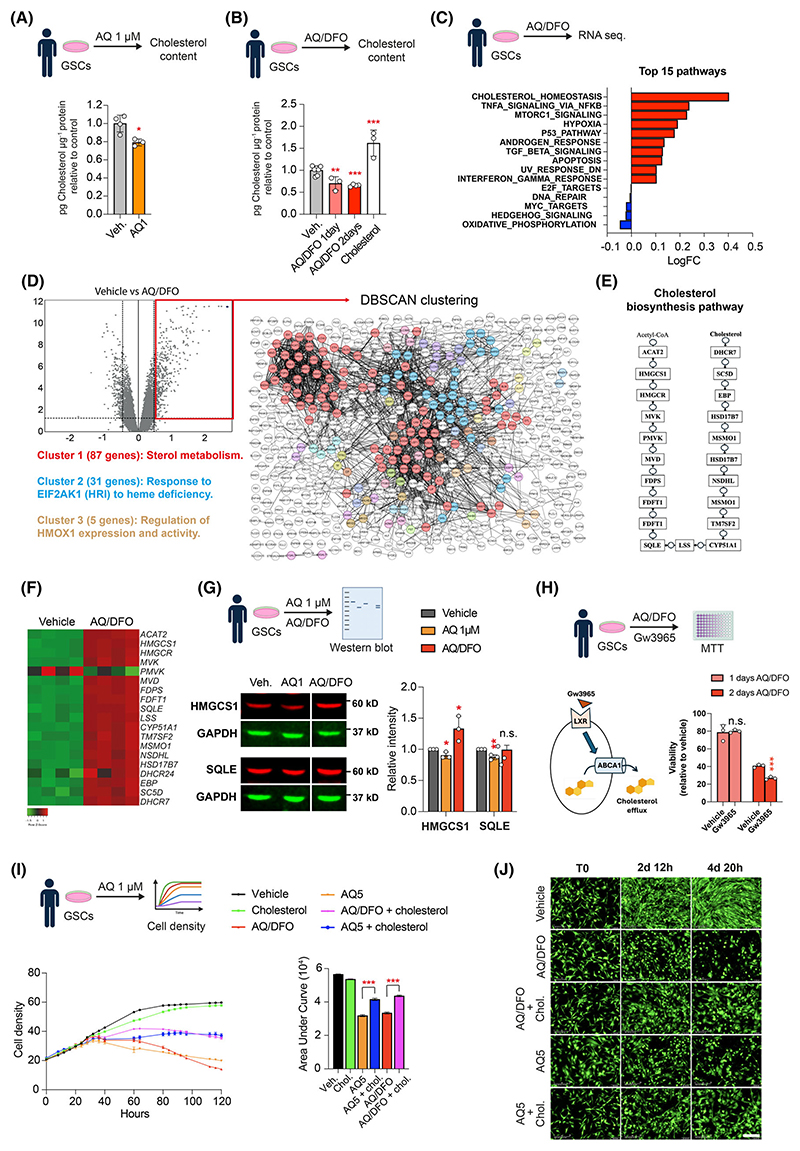
The combination of Adaptaquin and iron chelators specifically impairs cholesterol metabolism in glioma stem cells. (A) Measurement of cholesterol content in glioma stem cells (GSCs) treated with vehicle or 1 μM Adaptaquin (AQ) for 2 days (*n* = 3–4). (B) Measurement of cholesterol content in GSCs treated with vehicle or the combination of 1 μM Adaptaquin/10 μM Deferoxamine (AQ/DFO) for 1 and 2 days or with 20 μg·mL^−1^ cholesterol for 2 days (*n* = 3–7). (C) Top 15 regulated pathways in GSCs treated with AQ/DFO for 1 day. (D) Volcano plot showing dysregulated genes in GSCs treated with AQ/DFO treatment for 1 day with representation of Density-based Spatial Clustering of Applications with Noise (DBSCAN) clustering of upregulated genes (Log2FC > 0.5). (E) Schematic representation of the cholesterol biosynthesis pathway from Acetyl-CoA to cholesterol. (F) Heatmap highlighting genes of the cholesterol biosynthesis pathway regulated after treatment of GSCs with AQ/DFO for 1 day. (G) Western Blot analysis of 3-hydroxy-3-methylglutaryl-CoA synthase (HMGCS1) and squalene epoxidase (SQLE) in GSCs treated with 1 μM AQ or AQ/DFO for 1 day, with quantification relative to GAPDH (*n* = 3). (H) Viability of GSCs treated with Gw3965 or vehicle in combination with AQ/DFO for 1 and 2 days, measured by MTT (3-(4,5-dimethylthiazol-2-yl)-2,5 diphenyl tetrazolium bromide) assay (*n* = 3) with a schematic illustrating the mechanism of action of Gw3965 regulating cholesterol efflux. (I) Measurement of GSCs cell density over 5 days in cultures treated with AQ/DFO or 5 μM AQ, with or without 20 μg·mL^−1^ cholesterol supplementation (*n* = 4), with measure of Area Under Curve (AUC). (J) Fluorescence imaging of GFP in GSCs overexpressing GFP before treatment and after 2 and 4 days treatment with AQ/DFO or 5 μM AQ, with or without supplementation with 20 μg·mL^−1^ cholesterol. Data are represented as mean ± SEM. Significance using the two-tailed unpaired Student’s *t*-test for A, B, G, H and one-way ANOVA for I. *, *P* < 0.05; **, *P* < 0.01; ***, *P* < 0.001; ns, not significant. Scale bar: 200 μm.

To understand the functional impact of AQ on cholesterol synthesis in GSCs, we measured the cellular cholesterol level after 2 days of treatment with 1 μM AQ. This treatment led to a significant 20% reduction in cholesterol compared to vehicle control (Fig. 5A), correlating with decreased expression of cholesterol synthesis genes (Fig. 2A–E). Next, we examined the combined effect of AQ and DFO on cholesterol synthesis. Cholesterol content was measured after 1 and 2 days of AQ/DFO treatment, revealing a progressive decline, with a 40% reduction observed after 2 days (Fig. 5B). To further explore the link between cholesterol biosynthesis and AQ/DFO treatment, we conducted a new bulk RNA sequencing after 1 day of treatment (Fig. 5C). Cholesterol homeostasis emerged as the most upregulated pathway in gene ontology analysis, with increased expression of key cholesterol synthesis genes such as *MGCS1, SQLE, MSMO1*, and *INSIG1* (Fig. 5C–F and Fig. S4A). Moreover, DBSCAN clustering of upregulated genes highlighted sterol metabolism as the main cluster combining 87 genes of the 539 upregulated genes (Log2FC > 0.5) (Fig. 5D and Fig. S5). Western blot analysis confirmed the downregulation of HMGCS1 and SQLE proteins following 1 μM AQ treatment, aligning with transcriptomic data (Figs 5G and 2A–E). Interestingly, while SQLE protein levels remained unchanged, a modest increase in HMGCS1 protein was observed in cells treated with the combination AQ/DFO (Fig. 5G), suggesting a feedback regulation at the protein level.

Our findings demonstrate that combining AQ with DFO leads to an even greater depletion of cholesterol content in GSCs. We propose that this substantial reduction in cholesterol content triggers a compensatory feedback loop, upregulating cholesterol synthesis genes and enhancing activation of the biosynthetic pathway. In contrast, cholesterol content in human neurons remains similar with AQ/DFO treatment (Fig. S4B). As expected, quantitative RT-PCR analysis of transcripts involved in cholesterol synthesis such as *MGCS1, SQLE, MSMO1* and *INSIG1* showed none were affected by AQ/DFO treatment in neurons (Fig. S4C). These results show that AQ/DFO treatment impairs cholesterol synthesis specifically in GSCs.

Cholesterol homeostasis is known to be required for glioblastoma cell proliferation, migration and survival [13,15,16,34–38]. To understand whether cholesterol synthesis is involved in AQ/DFO-mediated GSC death, we first investigated whether the depletion of intracellular cholesterol sensitizes GSCs to AQ/DFO. To this end, we treated cells with Gw3965, a liver X receptor (LXRα/β) agonist known to promote cholesterol efflux by upregulating ABCA1. Gw3965 has been described as an anti-GBM agent due to its ability to lower cellular cholesterol and inhibit tumour cell growth [16,35]. Our results demonstrate that co-treatment with Gw3965 and AQ/DFO leads to a significant reduction in GSC viability after 48 h, indicating that AQ/DFO treatment further sensitizes cells to cholesterol depletion (Fig. 5H). Moreover, we performed cell growth analysis with the supplementation of extracellular cholesterol. As shown (Fig. 5I,J), 20 μg·mL^−1^ cholesterol supplementation was sufficient to partially protect GSCs against 5 μM AQ and AQ/DFO-mediated cell death. Taken together, these findings suggest that AQ/DFO treatment induces lethal stress in GSCs by disrupting cholesterol homeostasis.

### 3.7 Cholesterol protects GSCs against AQ/DFO-mediated mitochondrial dysfunction and production of reactive oxygen species

**Fig. 6:**
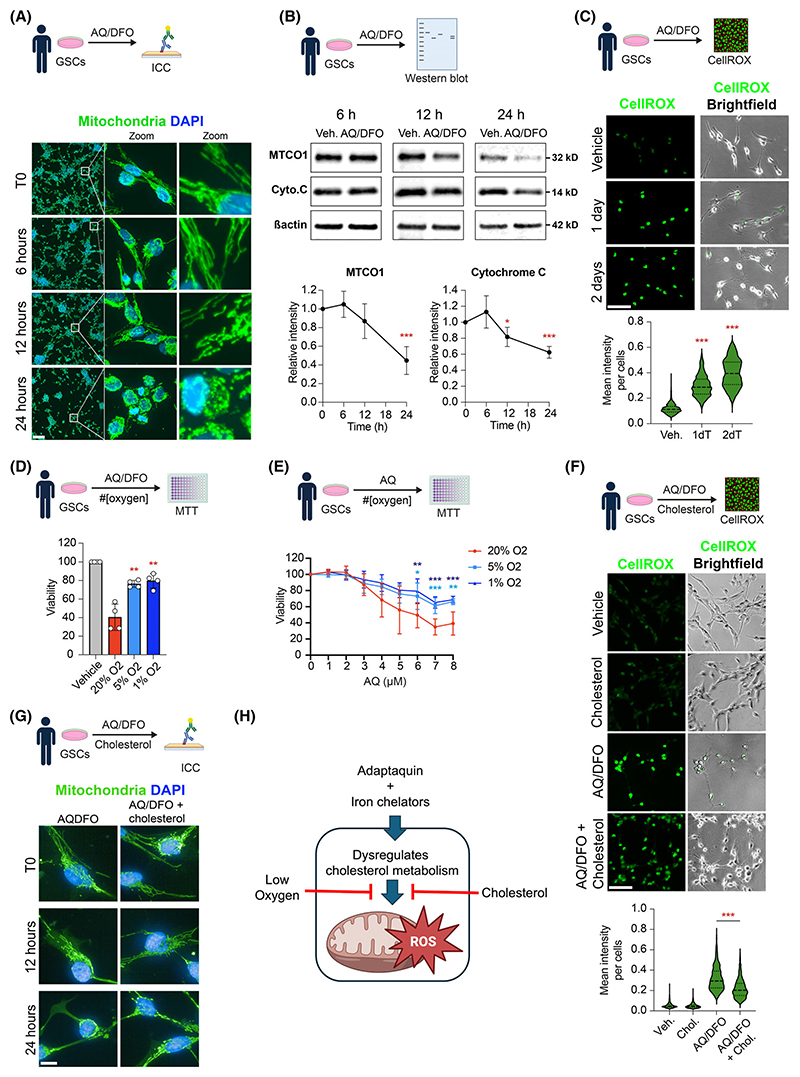
Cholesterol protects glioma stem cells against AQ/DFO-mediated mitochondrial disruption and protection of reactive oxygen species. Immunofluorescence imaging of mitochondria in glioma stem cells (GSCs) before treatment (T0) and after treatment with the combination of 1 μM Adaptaquin/10 μM Deferoxamine (AQ/DFO) for 6, 12 and 24 h, Magnified views of the regions outlined by white squares are shown. (B) Western Blot analysis of mitochondrial protein mitochondrially encoded cytochrome c oxidase I (MTCO1) and Cytochrome C in GSCs treated with AQ/DFO for 6, 12 and 24 h, with quantification relative to actin (*n* = 3–4). (C) Fluorescence imaging of CellROX fluorescent probes in GSCs treated with AQ/DFO for 1 and 2 days, with quantification (402–838 cells quantified over 3 independent replicates). (D) Viability of GSCs treated with AQ/DFO for 2 days and exposed to decreasing oxygen level, measured by MTT (3-(4,5-dimethylthiazol-2-yl)-2,5 diphenyl tetrazolium bromide) assay (*n* = 4). (E) Viability of GSCs treated with Adaptaquin (AQ) for 2 days and exposed to decreasing oxygen level, measured by MTT assay (*n* = 6). (F) Fluorescence imaging of CellROX fluorescent probes in GSCs treated with AQ/DFO with or without 20 μg·mL^−1^ cholesterol supplementation for 2 days, with quantification (814–2269 cells quantified over 3 independent replicates). (G) Immunofluorescence imaging of mitochondria in GSCs before treatment (T0) and after AQ/DFO treatment with or without 20 μg·mL^−1^ cholesterol supplementation for 12 and 24 h. (H) Schematic summarizing the effect of AQ/DFO on dysregulating cholesterol metabolism, leading to mitochondrial impairment and reactive oxygen species production. Data are represented as mean ± SEM. Significance using the two-tailed unpaired Student’s *t*-test for B, C, D, F and one-way ANOVA for E. *, *P* < 0.05; **, *P* < 0.01; ***, *P* < 0.001; ns, not significant. Scale bar: 100 μm for A, C, F and scale bar: 10 μm for G.

Our findings above indicated that iron and cholesterol supplementation could partially rescue the toxicity of AQ/DFO in GSCs. Cholesterol dysregulation can induce mitochondrial damage, alter membrane properties and electron transport chain (ETC) complexes, and generally affect mitochondrial health and function [39,40]. Similarly, iron is a crucial component of multiple ETC complexes, and disruptions in iron metabolism can impair ETC function, resulting in elevated production of reactive oxygen species (ROS) and mitochondrial dysfunction [41]. We assessed mitochondrial integrity in GSCs treated with AQ/DFO for 6, 12, or 24 h and found that this treatment compromised mitochondrial integrity as early as 12 h (Fig. 6A). Mitochondrial impairment was associated with a reduction in mitochondrial proteins MTCO1 and Cytochrome C (Fig. 6B). Additionally, we quantified oxidative stress in GSCs using a ROS-sensitive fluorescent probe, and after 24 h of AQ/DFO treatment, oxidative stress levels were significantly increased (Fig. 6C). Consistent with these observations, reducing ROS by lowering ambient oxygen levels to 5% or 1% (vis 20%) reduced AQ/DFO toxicity, both when administered alone and in combination with iron chelation (Fig. 6D,E). Importantly, cholesterol supplementation reduced oxidative stress in GSCs, as indicated by decreased fluorescence intensity from the ROS-sensitive probe (Fig. 6F), and provided partial protection against AQ/DFO-induced mitochondrial damage (Fig. 6G).

In summary, our results demonstrate that AQ/DFO treatment disrupts mitochondrial integrity and function in GSCs, leading to oxidative stress and cellular damage. The protective effect of cholesterol supplementation highlights the significance of cholesterol-mediated mitochondrial dysfunction as a critical endpoint of AQ/DFO GSC treatment (Fig. 6H).

## 4 Discussion

Adaptaquin (AQ) has been investigated as a neuroprotective drug in models of neurodegenerative diseases, including brain haemorrhage and Parkinson’s disease [23,24]. *In vivo*, AQ treatment enhances the survival of neurons and oligodendrocytes following hypoxic lesions in mice, while *in vitro*, AQ restores mitochondrial function and protects neurons from glutamate-induced cell death [22,24]. In this study, we demonstrate an additional effect of AQ to selectively induce cell death in proliferative progenitor cells, including neural stem cells, oligodendrocyte precursor cells, and patient-derived GSCs. Our evidence indicates the selective cytotoxicity of AQ reflects dysregulation of iron and cholesterol pathways essential for mitochondrial function in GSCs, while sparing neurons and other mature CNS cell types.

We chose AQ for this study based on its known dual properties as a neuroprotective agent that also targets PHD2, which is necessary for GSC survival and growth. Initial assessment revealed AQ’s cytotoxicity at higher doses in GSCs. Moreover, transcriptomic analysis showed upregulation of iron transport genes (e.g., *TFRC*) and downregulation of those involved in cholesterol biosynthesis. Combining AQ with iron chelation significantly enhanced its cytotoxic effects, and this could be rescued by elemental or heme-iron supplementation. This confirmed a state of enhanced functional iron dependence of AQ-treated GSCs. Because AQ does not act as a direct iron chelator [23], our findings indicate it modulates iron metabolism indirectly, consistent with prior findings in Schwann cells treated with AQ versus the iron chelator deferoxamine (DFO) [42]. Iron trafficking and storage proteins typically are regulated through the iron-responsive element (IRE) located within the mRNA for iron regulatory proteins (IRPs). Binding of iron to IRPs regulates translation or cleavage of mRNA targets. Reduced intracellular iron levels lead to upregulation of the iron transporter *TFRC*, while downregulating iron storage *FTL* and *FTH1*. Our data showed upregulation of *TFRC* followed by upregulation of *FTL* and *FTH1*, suggesting a regulatory mechanism independent of canonical iron-mediated IRP/IRE post-transcriptional control and likely driven at the transcriptional level. Specifically, we found that TFRC was upregulated, whereas FTL and FTH1 protein levels were reduced, indicating a decoupling between mRNA abundance and protein expression. This discrepancy suggests the involvement of non-canonical regulatory mechanisms that operate independently of the IRP/IRE system such as protein degradation pathways and post-transcriptional repression. Furthermore, the transcriptional regulation of TFRC is also known to be influenced by HIF1α via a Hypoxia Response Element (HRE) in its promoter region. However, our RNA-seq data revealed no significant activation of canonical HIF1α target genes (e.g., *VEGFA, BNIP3, GLUT1* and *PDK1*) (data not shown), indicating that the HIF1α pathway is not substantially engaged under our treatment conditions. Therefore, while we observed upregulation of TFRC, it is unlikely to be mediated by HIF1α. Targeting iron metabolism is a promising strategy in glioblastoma therapy, as these tumours are highly sensitive to iron availability. While the role of iron trafficking in glioblastoma progression remains incompletely understood and can have pro-survival and cytotoxic effects, blocking iron transport impairs glioblastoma growth in rodent models, and the overexpression of iron-related genes like TFRC and ferritins correlates with greater tumour aggressiveness and poorer patient survival [12,14].

An interesting implication of our findings is that heme is an alternative source of iron, and in this respect it is relevant that GSCs possess a heme transporter, which we have recently shown functions in iron transport in oligodendrocytes [43]. To prove a functional heme transporter in GSCs, we show here that they can take up the MRI-sensitive heme analogue MnPP as well as the fluorescent probe ZnMP.

Beyond iron metabolism, we identified cholesterol homeostasis as a critical pathway disrupted by AQ and AQ/DFO treatment in GSCs. Adaptaquin alone downregulates key cholesterol-related genes, including *INSIG1, HMGCS1, SQLE*, and *MSMO1*, while reducing cholesterol content by 20%. When combined with the iron chelator deferoxamine, cholesterol levels in GSCs decrease even further, reaching a 40% reduction. We hypothesize that this significant depletion triggers a compensatory feedback loop, leading to the upregulation of cholesterol synthesis genes and heightened activation of the cholesterol biosynthesis pathway. The ability of cancer cells to maintain cholesterol homeostasis underscores its critical role in cell survival and function. Notably, GSCs exhibit elevated cholesterol uptake, a feature associated with poor patient prognosis [13]. Previous studies have demonstrated the therapeutic potential of targeting cholesterol metabolism, with approaches such as statins and cholesterol derivatives effectively reducing glioblastoma viability and growth [15,16,35,44,45]. Our findings suggest that cholesterol depletion is a key mechanism underlying AQ/DFO-induced GSC death, providing a novel perspective for therapeutic intervention.

While AQ has been shown to exert neuroprotective effects via PHD2 inhibition and suppression of the ATF4 pro-death pathway in neurons [23,24], our findings indicate that these pathways play secondary roles in AQ’s toxicity against GSCs. Loss and gain-of-function experiments using siRNA and overexpression of PHD2 and HIF1α in GSCs revealed that AQ and AQ/DFO toxicity are independent of these pathways.

Similarly, while treatment with AQ in combination with DFO shows an upregulation of the ATF4 pathway in GSCs, blocking this pathway using the eIF2α inhibitor ISRIB did not protect the cells (Fig. S6). While these results suggest that AQ operates through other mechanisms, our transcriptomic analysis following 1 day of AQ/DFO treatment revealed no significant enrichment of gene ontology terms associated with fatty acid oxidation (GOBP: lipid oxidation) (Fig. S7), suggesting that lipid peroxidation is unlikely to be a transcriptionally regulated component of the cellular response to these treatments. Thus, further studies are indicated to explore lipid peroxidation and other potential non-transcriptional mechanisms contributing to GSC death. Steroid biosynthesis emerged as the most prominently dysregulated pathway following AQ treatment with minimal impact on glycolytic and other canonical HIF-driven processes (Fig. S8B).

Additional findings suggested that AQ disrupts cholesterol synthesis in a PHD2-independent manner, and that cholesterol depletion contributes to cytotoxicity. Cholesterol is essential for maintaining mitochondrial membrane structure and function, and its reduction can impair membrane fluidity, disrupt electron transport chain activity, and compromise ATP production [39–41]. The resulting metabolic stress may promote ROS accumulation and apoptotic signalling independently of transcriptional regulators such as PHD2 and HIF1α. Such mitochondrial vulnerability driven by cholesterol depletion might therefore amplify sensitivity to oxidative stress. This metabolic and mitochondrial targeting is particularly intriguing, as it aligns with emerging therapeutic strategies aimed at exploiting metabolic vulnerabilities by targeting non-oncogene addiction in cancer cells [28,29,46]. However, the precise molecular targets of AQ remain unknown and will require further investigation to clarify their role in glioblastoma therapy.

This study has focussed on the *in vitro* characterization of AQ using human cell lines to establish proof of concept for agents that can selectively kill GSCs while sparing neurons and maturing other CNS cell types. While an important question remains whether AQ would have *in vivo* efficacy, a key challenge in translating these findings is the complexity of the tumour microenvironment. For example, our results indicate that hypoxic conditions could protect GSCs from AQ-induced mitochondrial dysfunction, as low-oxygen levels reduced AQ toxicity. Indeed, RNA sequencing analysis of GSCs treated with AQ/DFO under hypoxic conditions revealed no dysregulation of cholesterol metabolism genes compared to normoxia, and cellular cholesterol levels remained unchanged after 2 days of AQ/DFO treatment in hypoxia (Fig. S8). These findings are the most consistent with the reduced oxidative stress associated with hypoxic conditions and the reduced impact of AQ on cholesterol metabolism in conditions of hypoxia. Additionally, cholesterol provided by astrocytes, microglia or other components of the microenvironment [47] could counteract the cholesterol depletion we observed *in vitro*, reducing AQ’s efficacy. However, our results also demonstrate that the combination of AQ with iron chelation does not significantly affect HIF regulation, a concern often associated with iron chelators, indicating a possible advantage for this therapeutic strategy. Further *in vivo* studies are needed to evaluate the therapeutic potential of AQ along with standard-of-care glioblastoma treatments such as temozolomide (TMZ). Because AQ has been shown to have neuroprotective effects, it might assuage neurotoxic agents like TMZ and/or radiation to protect healthy neural tissue while targeting GSCs. However, our findings also indicate that oligodendrocyte precursor cells (OPCs) are particularly sensitive to AQ. While this may raise concerns during development, short-term OPC depletion in the adult brain is likely reversible [48,49]. Future *in vivo* studies would explore the impact of AQ on progenitor cell populations and consider treatment duration to better define its therapeutic potential and safety.

## 5 Conclusion

Our findings demonstrate that Adaptaquin (AQ) selectively induces death of glioma stem cells (GSCs) and proliferative cells, while sparing differentiated cell populations such as mature neurons, oligodendrocytes and astrocytes. Transcriptomic analyses revealed that AQ disrupts key metabolic pathways, notably iron and cholesterol homeostasis. Indeed, iron chelation enhanced AQ cytotoxicity, leading to marked depletion of cellular cholesterol, compromising mitochondrial integrity and increasing reactive oxygen species (ROS) levels. Together, these results underscore the therapeutic potential of AQ in selectively targeting GSCs metabolism by inducing iron and cholesterol dependency states, which have been the focus of non-oncogene addiction therapeutic strategies.

## Supplementary Material

Supporting Information

## Data Availability

The data generated in this study are available upon request from the corresponding author, DHR (david.rowitch@cshs.org).
