# Supplementary material for: Adaptaquin is selectively toxic to glioma stem cells through disruption of iron and cholesterol metabolism
Source: Mol Oncol. 2025 Sep 21;20(2):307–30. doi: 10.1002/1878-0261.70128 (PMC7618758; doi:10.1002/1878-0261.70128)
Supplement: Supplementary file 1 — Fig. S1. Neurons from human cerebral organoids are resistant to Adaptaquin treatment. Fig. S2. A functional heme transporter in glioma stem cells. Fig. S3. Regulation of prolyl hydroxylases and hypoxia inducible factors. Fig. S4. Regulation of cholesterol pathway in iPSC‐derived neurons treated with the combination of Adaptaquin and deferoxamine. Fig. S5. List of clusters from DBSCAN clustering of regulated genes after AQ/DFO treatment in glioma stem cells. Fig. S6. AQ‐mediated glioma stem cell death is independent of ATF4. Fig. S7. Adaptaquin do not regulates genes associated with fatty acid oxidation in glioma stem cells. Fig. S8. Hypoxia prevent Adaptaquin‐mediated cholesterol dysregulation in glioma stem cells. [file MOL2-20-307-s001.zip › mol270128-sup-0005-SupplementaryFigureS5.pdf]

A

color	cluster Id	gene count	description
	Cluster 1	87	+ Sterol metabolism
	Cluster 2	31	Response of EIF2AK1 (HRI) to heme deficiency
	Cluster 3	5	+ Regulation of HMOX1 expression and activity
	Cluster 4	5	+ Progesterone receptor signaling pathway
	Cluster 5	4	Circadian rhythm
	Cluster 6	3	+ Autophagy of nucleus
	Cluster 7	3	HFE-transferrin receptor complex
	Cluster 8	2	+ Netrin mediated repulsion signals
	Cluster 9	2	non-Langerhans-cell histiocytosis, and Alveolar lamellar body membrane
	Cluster 10	2	Regulation of signaling by NODAL, and Glycoprotein hormones
	Cluster 11	2	+ Lipoprotein lipase activity
	Cluster 12	2	NPC1, TMEM97
	Cluster 13	2	Mixed, incl. ARHGEF1-like, PH domain, and Rho protein GDP-dissociation inhibitor
	Cluster 14	2	Mixed, incl. PRA1 family protein, and Snail allergy
	Cluster 15	2	+ Vitamin C
	Cluster 16	2	Positive regulation of blood-brain barrier permeability, and Calcium-independent cell...
	Cluster 17	2	ANK2, ITPR1
	Cluster 18	2	Dopamine receptor signaling pathway
	Cluster 19	2	Osteopetrosis
	Cluster 20	2	+ One carbon pool by folate
	Cluster 21	2	Glycine, serine and threonine metabolism
	Cluster 22	2	Mixed, incl. PET Domain, and Dishevelled family
	Cluster 23	2	Myotonic dystrophy type 1, and CELF-3/4/5/6, RNA recognition motif 1
	Cluster 24	2	beta-1,3-galactosyltransferase activity
	Cluster 25	2	Calcitonin-like ligand receptors
	Cluster 26	2	+ Neutral amino acid:sodium symporter activity
	Cluster 27	2	Integrin
	Cluster 28	2	GPR1, RARRES2

B

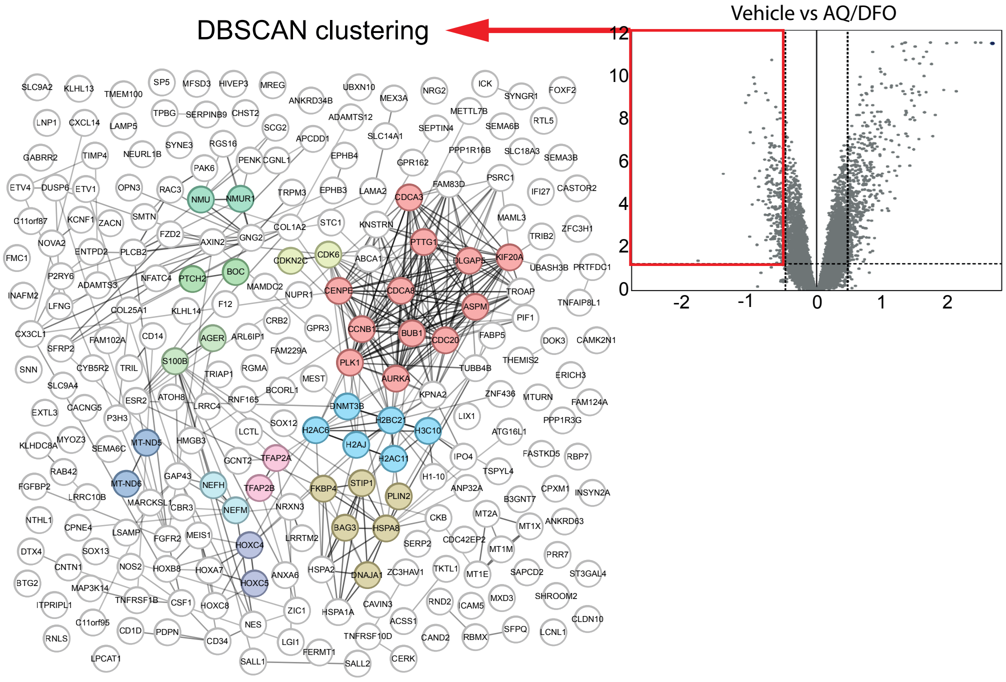

C

color	cluster Id	gene count	description
	Cluster 1	12	Regulation of mitotic sister chromatid separation
	Cluster 2	6	+ DNA methylation
	Cluster 3	6	+ HSP90 chaperone cycle for steroid hormone receptors (SHR) in the presence of l...
	Cluster 4	2	+ Oncogene Induced Senescence
	Cluster 5	2	Nevoid basal cell carcinoma syndrome, and Hedgehog protein
	Cluster 6	2	+ Advanced glycosylation endproduct receptor signaling
	Cluster 7	2	Neuromedin U binding, and Neuromedin U, amidation site
	Cluster 8	2	+ Neurofilament bundle assembly
	Cluster 9	2	MELAS syndrome
	Cluster 10	2	Homeobox protein, antennapedia type, and Homeobox protein HXA9/HXB9/HXC9
	Cluster 11	2	Negative regulation of activity of TFAP2 (AP-2) family transcription factors
