# Supplementary material for: Adaptaquin is selectively toxic to glioma stem cells through disruption of iron and cholesterol metabolism
Source: Mol Oncol. 2025 Sep 21;20(2):307–30. doi: 10.1002/1878-0261.70128 (PMC7618758; doi:10.1002/1878-0261.70128)
Supplement: Supplementary file 1 — Fig. S1. Neurons from human cerebral organoids are resistant to Adaptaquin treatment. Fig. S2. A functional heme transporter in glioma stem cells. Fig. S3. Regulation of prolyl hydroxylases and hypoxia inducible factors. Fig. S4. Regulation of cholesterol pathway in iPSC‐derived neurons treated with the combination of Adaptaquin and deferoxamine. Fig. S5. List of clusters from DBSCAN clustering of regulated genes after AQ/DFO treatment in glioma stem cells. Fig. S6. AQ‐mediated glioma stem cell death is independent of ATF4. Fig. S7. Adaptaquin do not regulates genes associated with fatty acid oxidation in glioma stem cells. Fig. S8. Hypoxia prevent Adaptaquin‐mediated cholesterol dysregulation in glioma stem cells. [file MOL2-20-307-s001.zip › Supplementary information.docx]

**Supplementary Figure S1. Neurons from human cerebral organoids are resistant to Adaptaquin treatment.**

**A-B**, Immunofluorescence imaging of of the neuronal markers Neurofilament-M (NFM) (A) and Beta-3 tubulin (TUJ1) (B) together with DAPI of 10-weeks-old hESC-derived cerebral organoids after 2 days of treatment with 7 µM Adaptaquin. Scale bar: 50µm

**Supplementary Figure S2. A functional heme transporter in glioma stem cells.**

**A**, Schematic illustrating the uptake of Transferrin-bound iron in glioma stem cells (GSCs) through transferrin receptor (TFRC) and the uptake of heme through an unknown transporter. **B**, Chemical structures of heme, the heme analogue hemin, the inhibitor of heme oxygenase 1 and 2 (HMOX1/2) and fluorescent Zinc(II) Mesoporphyrin IX (ZnMP) and the MRI-sensitive Manganese(III) Protoporphyrin IX chloride (MnPP). **C**, Schematic illustrating the inhibition by ZnMP of the oxidative degradation of hemin by HMOX1/2. **D**, Viability of GSCs treated with the combination of 1 µM Adaptaquin / 10 µM Deferoxamine (AQ/DFO) in combination with Hemin and ZnMP (n=4). **E-F**, Viability of GSCs (E) or iPSC-derived neurons (F) treated with Deferoxamine (DFO) with or without 15 µM hemin for 2 days, measured by MTT (3-[4,5-dimethylthiazol-2-yl]-2,5 diphenyl tetrazolium bromide) assay (n=3). **G**, Fluorescence imaging of ZnMP together with DAPI of GSCs treated with ZnMP, with quantification (204-381 cells quantified over 3 independent replicates). **H**, Fluorescence imaging of GSCs and HEK cell pellets treated with DMSO or ZnMP, with quantification (n=3-4). **I**, Relaxation rate (R_1_) map of serial 1:2 dilutions of MnPP from 0-1.6 mM in 10% DMSO. The slope of this graph (relaxation rate, R_1_ vs MnPP concentration) determines the relaxivity, r_1_, of MnPP, which allows linking the relaxation rate, R_1_, to the concentration of MnPP, hence making measurements quantifiable (n=2). **J**, Relaxation rate (R_1_) map of GSCs and HEK cell pellets treated with DMSO or MnPP, with quantification (n=3). Data are represented as mean ± SEM. Significance using the two-tailed unpaired student *t* test for D, G, H, J and one-way ANOVA for E and F. *, P<0.05; **, P<0.01; ***, P<0.001; ns, not significant. Scale bar: 100µm

**Supplementary Figure S3. Regulation of prolyl hydroxylases and hypoxia inducible factors.**

**A-B**, Quantification of *PHD1* (A) and *PHD3* (B) expression level in glioma stem cells (GSCs) treated with vehicle or with the combination of 1 µM Adaptaquin / 10 µM Deferoxamine (AQ/DFO) for 1 day and transfected with siRNA control or siRNA targeting *PHD2*, measured by RT-qPCR relate to actin (n=2-3). **C-D**, Quantification of *PHD1* (C) and *PHD3* (D) expression level in GSCs overexpressing GFP (GFPover) and PHD2 (PHD2over), measured by RT-qPCR relative to actin (n=3). **E-F**, Quantification of *HIF1b* (E) and *HIF2a* (F) expression level in GSCs transfected with siRNA control or siRNA targeting *HIF1α*, measured by RT-qPCR relate to actin (n=2-3). Data are represented as mean ± SEM. Significance using the two-tailed unpaired student *t* test. *, P<0.05; **, P<0.01; ***, P<0.001; ns, not significant.

**Supplementary Figure S4**. **Regulation of cholesterol pathway in iPSC-derived neurons treated with the combination of Adaptaquin and deferoxamine.**

**A**, Quantification of *3-hydroxy-3-methylglutaryl-CoA synthase (HMGCS1), squalene epoxidase (SQLE), methylsterol monooxygenase (MSMO1), insulin induced gene (INSIG1)* expression level in glioma stem cells (GSCs) treated with AQ/DFO for 1 day, measured by RT-qPCR relative to actin (n=4). **B**, Measurement of cholesterol content in human iPSC-derived neurons treated with the combination of 1 µM Adaptaquin / 10 µM Deferoxamine (AQ/DFO) for 2 days (n=3). **C**, Quantification of *HMGCS1, SQLE, MSMO1, INSIG1* expression level in human iPSC-derived neurons treated with AQ/DFO for 1 day, measured by RT-qPCR relative to actin (n=4). Data are represented as mean ± SEM. Significance using the two-tailed unpaired student *t* test. *, P<0.05; **, P<0.01; ***, P<0.001; ns, not significant.

**Supplementary Figure S5. List of clusters from DBSCAN clustering of regulated genes after AQ/DFO treatment in glioma stem cells.**

**A**, List of the 28 clusters description from Density-based Spatial Clustering of Applications with Noise (DBSCAN) clustering of upregulated genes after treatment with the combination of 1 µM Adaptaquin / 10 µM Deferoxamine (AQ/DFO) in glioma stem cells (GSCs) for 1 day. The DBSCAN clustering is represented in figure 6 D. **B-C**, DBSCAN clustering of downregulated genes (highlighted by the red square; Log2FC > 0.5) after 1 day treatment with the combination of 1 µM Adaptaquin and 10 µM Deferoxamine (AQ/DFO) (B), with the list of clusters description (C).

**Supplementary Figure S6. AQ-mediated glioma stem cell death is independent of ATF4.**

**A**, Heatmap representation of activating transcription factor-4 (ATF4) related genes expression levels in glioma stem cells (GSCs) after 1 day of treatment with the combination of 1 µM Adaptaquin and 10 µM Deferoxamine (AQ/DFO). **B**, Network node representation of proteins encoded by ATF4-related genes altered in GSCs after 1 day of AQ/DFO treatment, showing known and predicted interaction between them. **C**, Schematic representing activation of pro-apoptotic genes activation induced by endoplasmic reticulum (ER) stress through ATF4 pathway and its inhibition by integrated stress response inhibitor (ISRIB). **D**, Quantification of *ATF4*, DNA damage inducible transcript 3(*DDIT3)*, tribbles pseudokinase 3 (*TRIB3)* expression levels in GSCs treated with AQ/DFO with or without ISRIB inhibition, measured by RT-qPCR relative to actin (n=3-4). **E**, Measurement of GSCs cell density over 5 days in cultures treated with AQ/DFO with or without ISRIB inhibition (n=4). Data are represented as mean ± SEM. Significance using the two-tailed unpaired student *t* test. ***, P<0.001.

**Supplementary Figure S7**. **Adaptaquin do not regulates genes associated with fatty acid oxidation in glioma stem cells.**

**A**, Plot showing the distribution of gene ontology biological process (GOBP): Fatty acid oxidation (GO_0019395) gene set after 1 day treatment with the combination 1 µM Adaptaquin / 10 µM Deferoxamine (AQ/DFO) compared to vehicle-treated glioma stem cells (GSCs). **B**, Volcano plot representation showing differentially expressed genes with genes from the fatty acid oxidation processes (GOBP: fatty acid oxidation) highlighted in blue.

**Supplementary Figure S8**. **Hypoxia prevent Adaptaquin-mediated cholesterol dysregulation in glioma stem cells.**

**A**, Glioma stem cells (GCSs) were treated with 1 µM Adaptaquin / 10 µM Deferoxamine (AQ/DFO) for 1 day in hypoxia (1% O_2_) and analysed by RNA sequencing. **B**, KEGG activation matrix provides a comparative view of pathway activity levels across treatment conditions for GSCs treated with AQ/DFO for 1 day in hypoxia and normoxia (circle size represents relative pathway activation strength and the colour scale reflects upregulation (red) or downregulation (blue). **C**, Raw read counts expressed as counts per million (CPM) for the genes transferrin receptor (*TFRC), squalene epoxidase (SQLE), 3-hydroxy-3-methylglutaryl-CoA synthase (HMGCS1), methylsterol monooxygenase (MSMO1), insulin induced gene (INSIG1)* obtained from the RNA sequencing of GSCs treated with AQ/DFO for 1 day in normoxia or hypoxia. **D**, Measurement of cholesterol content in GSCs treated with vehicle or AQ/DFO for 2 days in hypoxia (n=4). Data are represented as mean ± SEM. Significance using the two-tailed unpaired student *t* test. ***, P<0.001; ns, not significant.
