# Supplementary figures and images for: Adaptaquin is selectively toxic to glioma stem cells through disruption of iron and cholesterol metabolism
Source: Mol Oncol. 2025 Sep 21;20(2):307–30. doi: 10.1002/1878-0261.70128 (PMC7618758; doi:10.1002/1878-0261.70128)

**A**

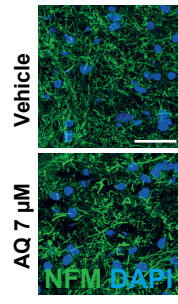

**B**

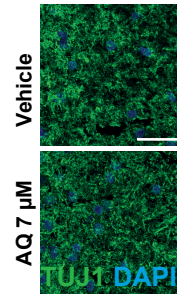

Supplement: Supplementary file 1 — Fig. S1. Neurons from human cerebral organoids are resistant to Adaptaquin treatment. Fig. S2. A functional heme transporter in glioma stem cells. Fig. S3. Regulation of prolyl hydroxylases and hypoxia inducible factors. Fig. S4. Regulation of cholesterol pathway in iPSC‐derived neurons treated with the combination of Adaptaquin and deferoxamine. Fig. S5. List of clusters from DBSCAN clustering of regulated genes after AQ/DFO treatment in glioma stem cells. Fig. S6. AQ‐mediated glioma stem cell death is independent of ATF4. Fig. S7. Adaptaquin do not regulates genes associated with fatty acid oxidation in glioma stem cells. Fig. S8. Hypoxia prevent Adaptaquin‐mediated cholesterol dysregulation in glioma stem cells. [file MOL2-20-307-s001.zip › mol270128-sup-0001-SupplementaryFigureS1.pdf]

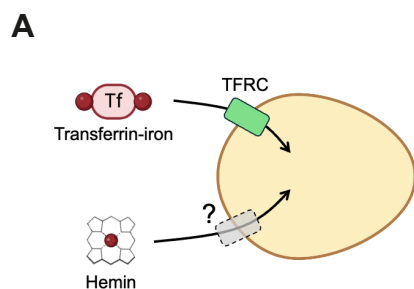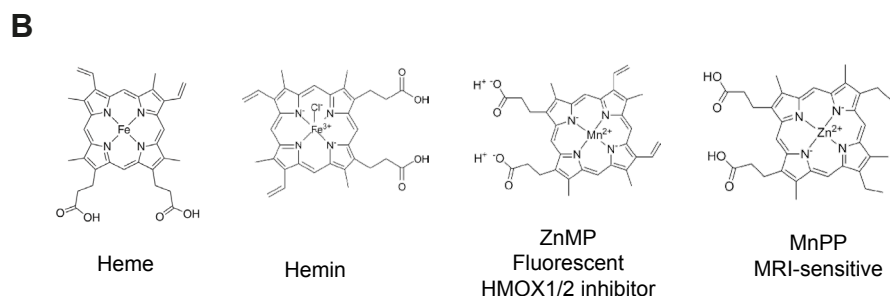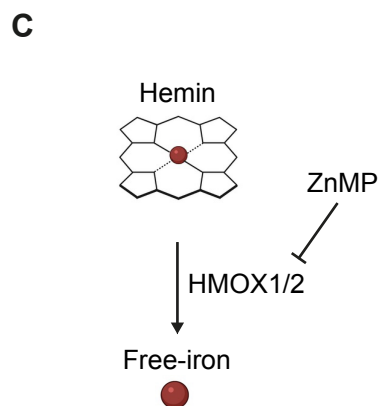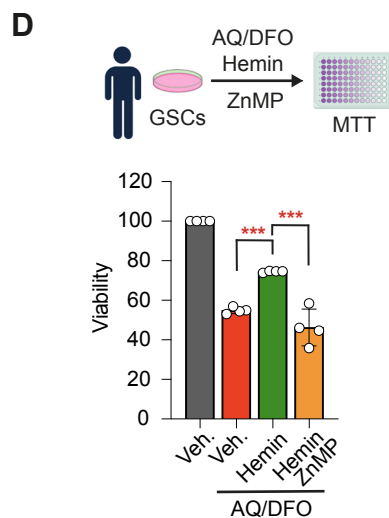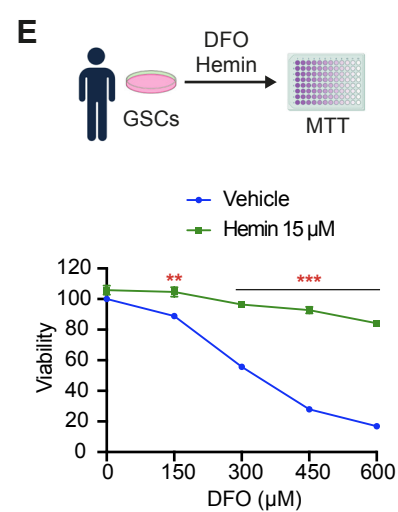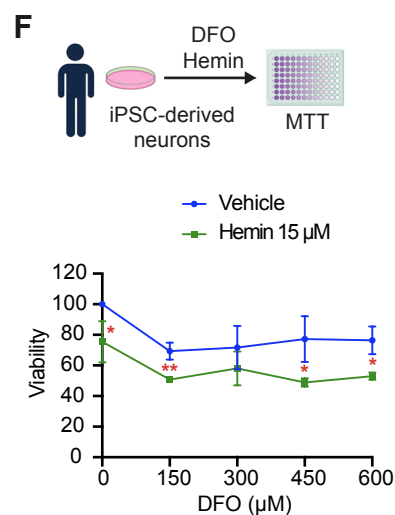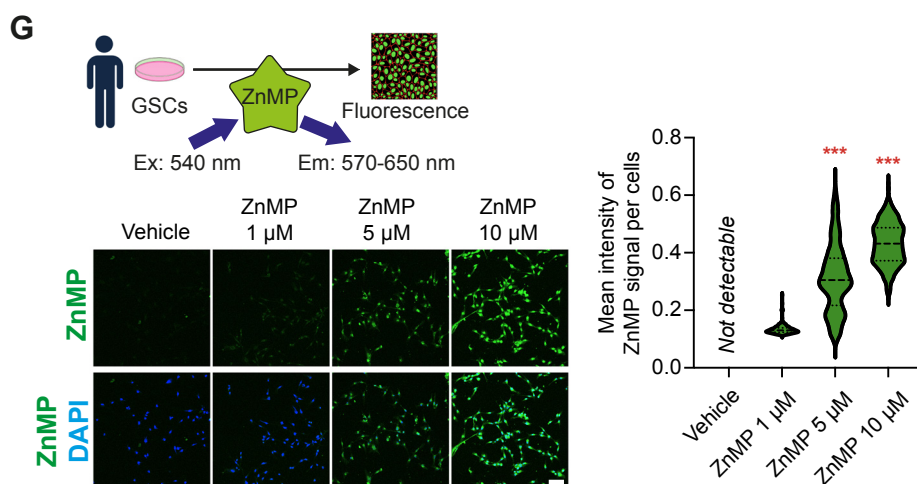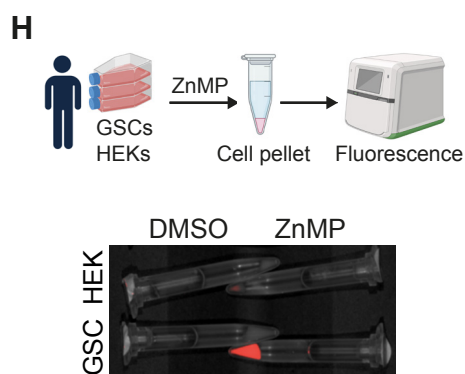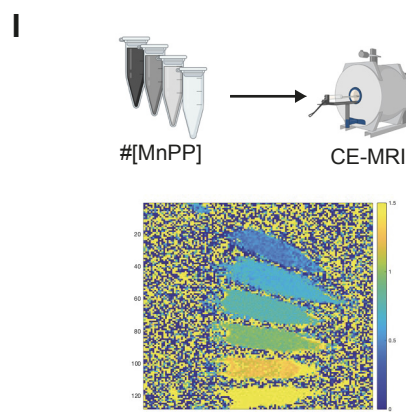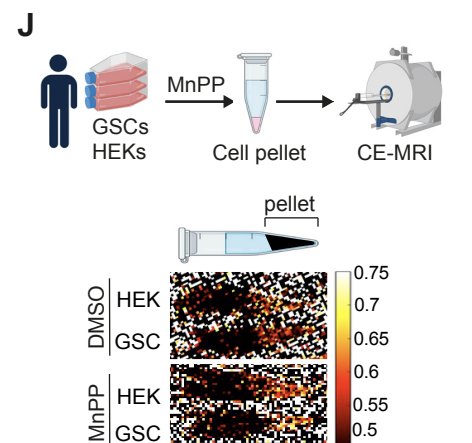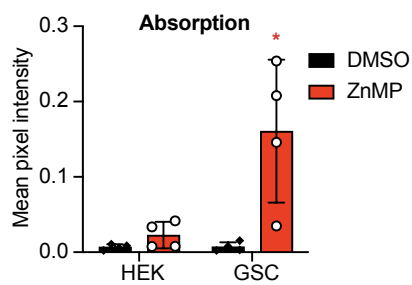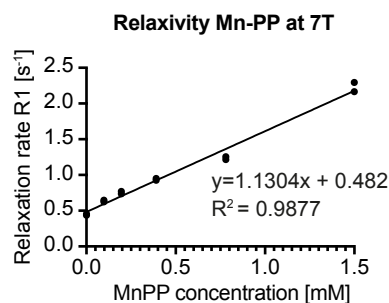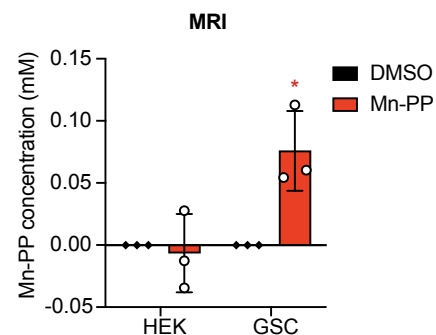

Supplement: Supplementary file 1 — Fig. S1. Neurons from human cerebral organoids are resistant to Adaptaquin treatment. Fig. S2. A functional heme transporter in glioma stem cells. Fig. S3. Regulation of prolyl hydroxylases and hypoxia inducible factors. Fig. S4. Regulation of cholesterol pathway in iPSC‐derived neurons treated with the combination of Adaptaquin and deferoxamine. Fig. S5. List of clusters from DBSCAN clustering of regulated genes after AQ/DFO treatment in glioma stem cells. Fig. S6. AQ‐mediated glioma stem cell death is independent of ATF4. Fig. S7. Adaptaquin do not regulates genes associated with fatty acid oxidation in glioma stem cells. Fig. S8. Hypoxia prevent Adaptaquin‐mediated cholesterol dysregulation in glioma stem cells. [file MOL2-20-307-s001.zip › mol270128-sup-0002-SupplementaryFigureS2.pdf]

**A**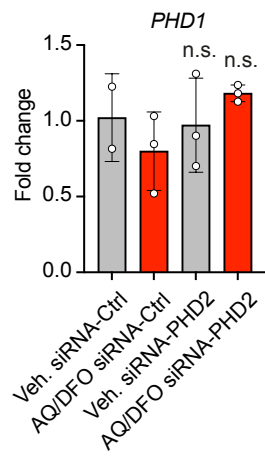**B**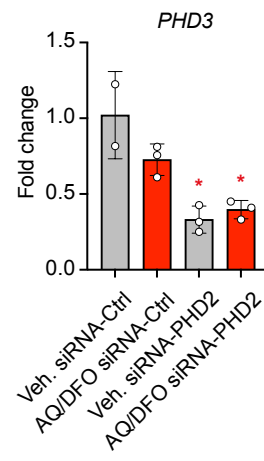**C**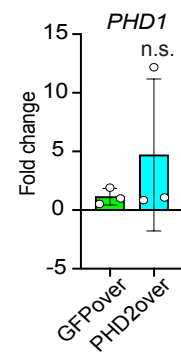**D**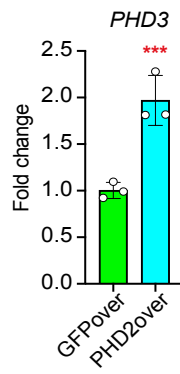**E**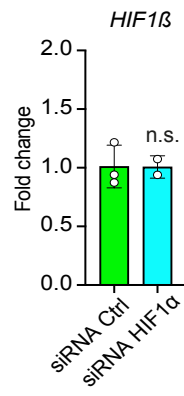**F**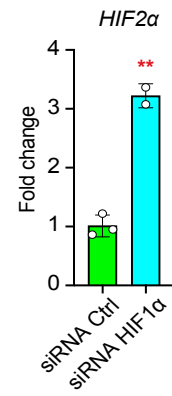

Supplement: Supplementary file 1 — Fig. S1. Neurons from human cerebral organoids are resistant to Adaptaquin treatment. Fig. S2. A functional heme transporter in glioma stem cells. Fig. S3. Regulation of prolyl hydroxylases and hypoxia inducible factors. Fig. S4. Regulation of cholesterol pathway in iPSC‐derived neurons treated with the combination of Adaptaquin and deferoxamine. Fig. S5. List of clusters from DBSCAN clustering of regulated genes after AQ/DFO treatment in glioma stem cells. Fig. S6. AQ‐mediated glioma stem cell death is independent of ATF4. Fig. S7. Adaptaquin do not regulates genes associated with fatty acid oxidation in glioma stem cells. Fig. S8. Hypoxia prevent Adaptaquin‐mediated cholesterol dysregulation in glioma stem cells. [file MOL2-20-307-s001.zip › mol270128-sup-0003-SupplementaryFigureS3.pdf]

**A**

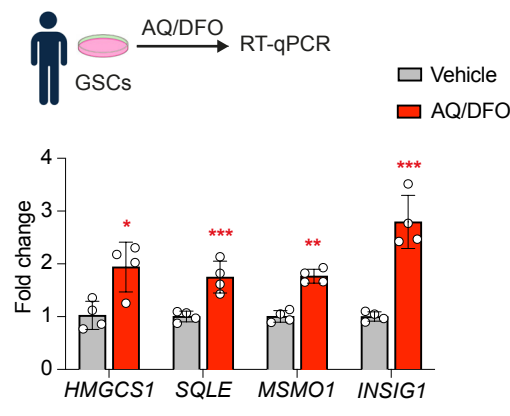

**B**

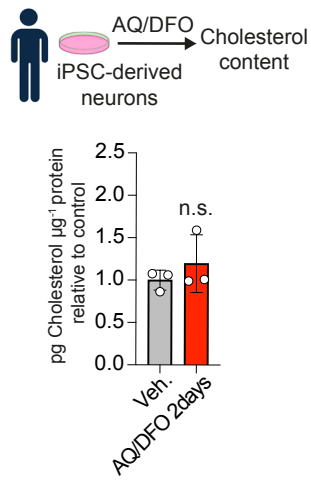

**C**

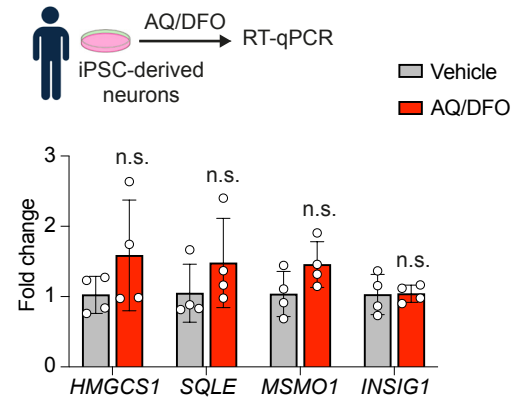

Supplement: Supplementary file 1 — Fig. S1. Neurons from human cerebral organoids are resistant to Adaptaquin treatment. Fig. S2. A functional heme transporter in glioma stem cells. Fig. S3. Regulation of prolyl hydroxylases and hypoxia inducible factors. Fig. S4. Regulation of cholesterol pathway in iPSC‐derived neurons treated with the combination of Adaptaquin and deferoxamine. Fig. S5. List of clusters from DBSCAN clustering of regulated genes after AQ/DFO treatment in glioma stem cells. Fig. S6. AQ‐mediated glioma stem cell death is independent of ATF4. Fig. S7. Adaptaquin do not regulates genes associated with fatty acid oxidation in glioma stem cells. Fig. S8. Hypoxia prevent Adaptaquin‐mediated cholesterol dysregulation in glioma stem cells. [file MOL2-20-307-s001.zip › mol270128-sup-0004-SupplementaryFigureS4.pdf]

**A**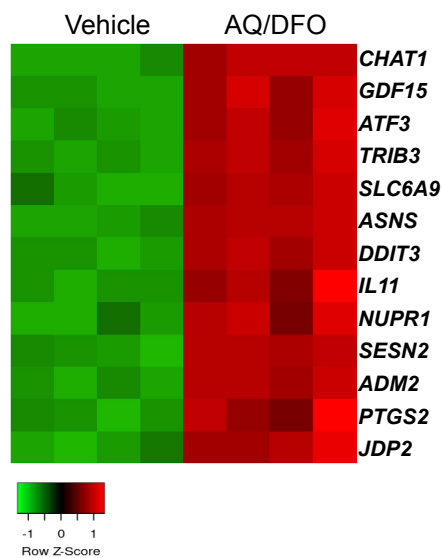**B**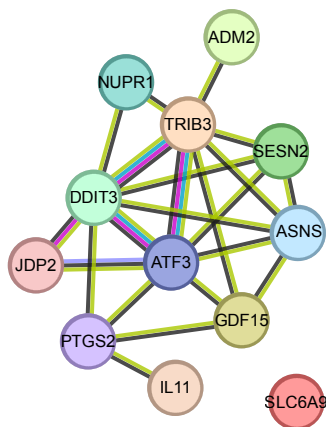**C**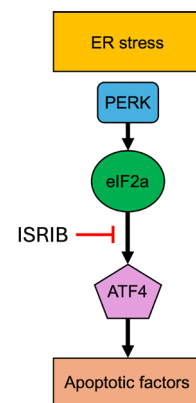**D**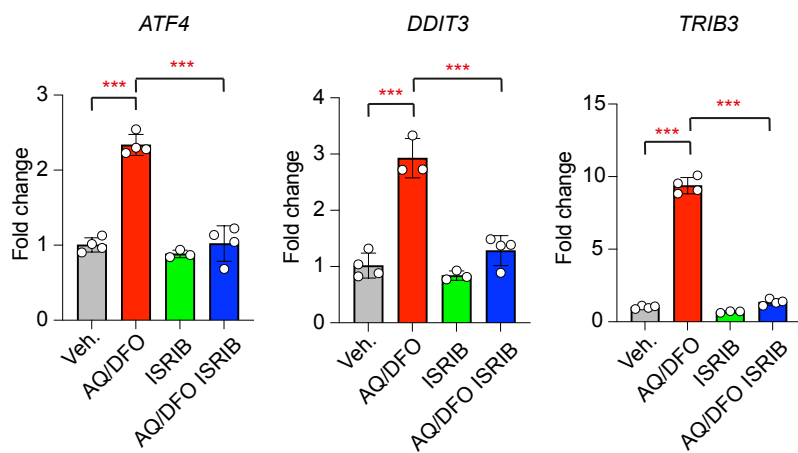**E**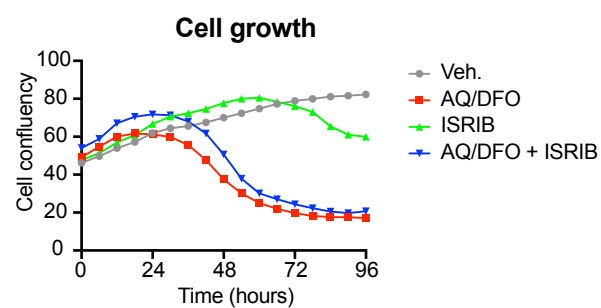

Supplement: Supplementary file 1 — Fig. S1. Neurons from human cerebral organoids are resistant to Adaptaquin treatment. Fig. S2. A functional heme transporter in glioma stem cells. Fig. S3. Regulation of prolyl hydroxylases and hypoxia inducible factors. Fig. S4. Regulation of cholesterol pathway in iPSC‐derived neurons treated with the combination of Adaptaquin and deferoxamine. Fig. S5. List of clusters from DBSCAN clustering of regulated genes after AQ/DFO treatment in glioma stem cells. Fig. S6. AQ‐mediated glioma stem cell death is independent of ATF4. Fig. S7. Adaptaquin do not regulates genes associated with fatty acid oxidation in glioma stem cells. Fig. S8. Hypoxia prevent Adaptaquin‐mediated cholesterol dysregulation in glioma stem cells. [file MOL2-20-307-s001.zip › mol270128-sup-0006-SupplementaryFigureS6.pdf]

**A**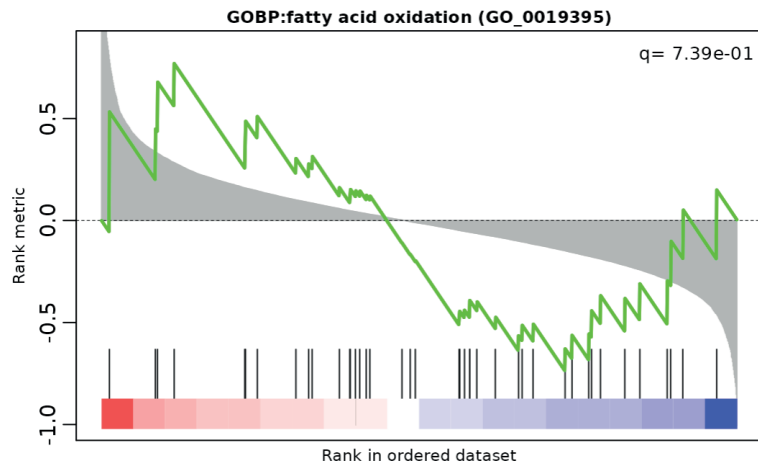**B**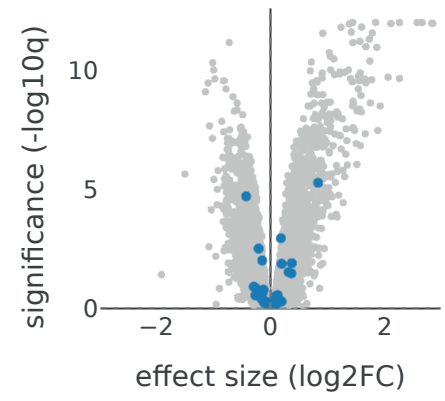

Supplement: Supplementary file 1 — Fig. S1. Neurons from human cerebral organoids are resistant to Adaptaquin treatment. Fig. S2. A functional heme transporter in glioma stem cells. Fig. S3. Regulation of prolyl hydroxylases and hypoxia inducible factors. Fig. S4. Regulation of cholesterol pathway in iPSC‐derived neurons treated with the combination of Adaptaquin and deferoxamine. Fig. S5. List of clusters from DBSCAN clustering of regulated genes after AQ/DFO treatment in glioma stem cells. Fig. S6. AQ‐mediated glioma stem cell death is independent of ATF4. Fig. S7. Adaptaquin do not regulates genes associated with fatty acid oxidation in glioma stem cells. Fig. S8. Hypoxia prevent Adaptaquin‐mediated cholesterol dysregulation in glioma stem cells. [file MOL2-20-307-s001.zip › mol270128-sup-0007-SupplementaryFigureS7.pdf]

A

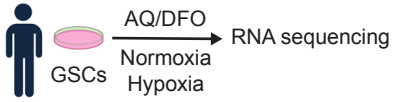

B

## KEGG activation matrix

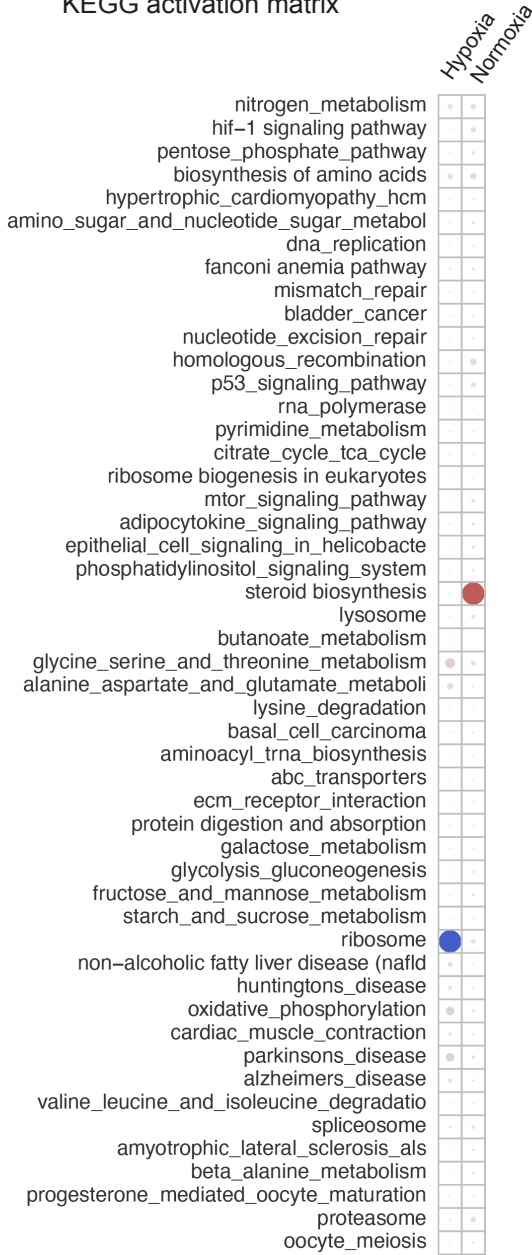

C

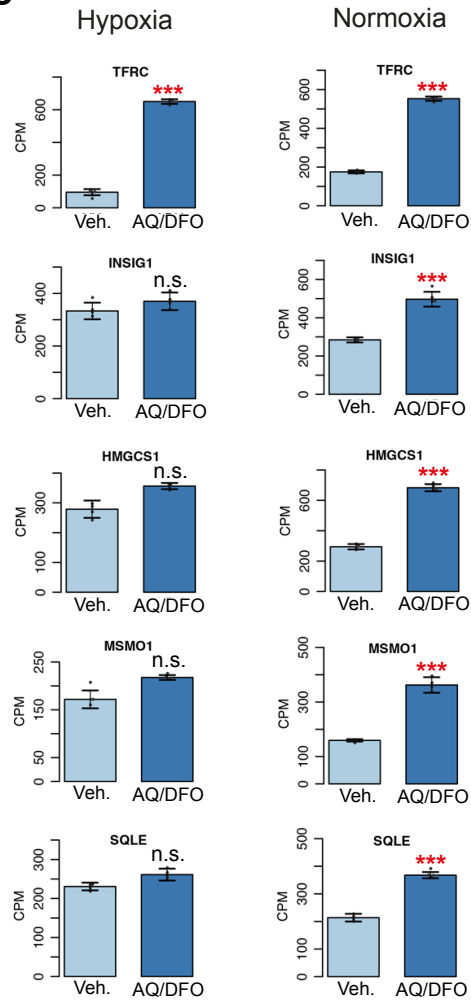

D

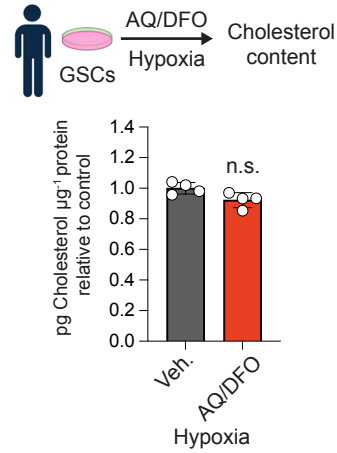

Supplement: Supplementary file 1 — Fig. S1. Neurons from human cerebral organoids are resistant to Adaptaquin treatment. Fig. S2. A functional heme transporter in glioma stem cells. Fig. S3. Regulation of prolyl hydroxylases and hypoxia inducible factors. Fig. S4. Regulation of cholesterol pathway in iPSC‐derived neurons treated with the combination of Adaptaquin and deferoxamine. Fig. S5. List of clusters from DBSCAN clustering of regulated genes after AQ/DFO treatment in glioma stem cells. Fig. S6. AQ‐mediated glioma stem cell death is independent of ATF4. Fig. S7. Adaptaquin do not regulates genes associated with fatty acid oxidation in glioma stem cells. Fig. S8. Hypoxia prevent Adaptaquin‐mediated cholesterol dysregulation in glioma stem cells. [file MOL2-20-307-s001.zip › mol270128-sup-0008-SupplementaryFigureS8.pdf]
